# Mitochondrial fatty acid oxidation drives senescence

**DOI:** 10.1126/sciadv.ado5887

**Published:** 2024-10-25

**Authors:** Shota Yamauchi, Yuki Sugiura, Junji Yamaguchi, Xiangyu Zhou, Satoshi Takenaka, Takeru Odawara, Shunsuke Fukaya, Takao Fujisawa, Isao Naguro, Yasuo Uchiyama, Akiko Takahashi, Hidenori Ichijo

**Affiliations:** ^1^ Laboratory of Cell Signaling, Graduate School of Pharmaceutical Sciences, University of Tokyo, Bunkyo-ku, Tokyo 113-0033, Japan.; ^2^ Division of Cellular Senescence, Cancer Institute, Japanese Foundation for Cancer Research, Koto-ku, Tokyo 135-8550, Japan.; ^3^ Center for Cancer Immunotherapy and Immunobiology, Kyoto University Graduate School of Medicine, Kyoto, Kyoto 606-8507, Japan.; ^4^ Laboratory of Morphology and Image Analysis, Biomedical Research Center, Juntendo University Graduate School of Medicine, Bunkyo-ku, Tokyo 113-8421, Japan.; ^5^ Department of Cellular and Molecular Neuropathology, Juntendo University Graduate School of Medicine, Bunkyo-ku, Tokyo 113-8421, Japan.; ^6^ Cell Signaling and Stress Responses Laboratory, Advanced Research Institute, Tokyo Medical and Dental University, Chiyoda-ku, Tokyo 101-0062, Japan.

## Abstract

Cellular senescence is a stress-induced irreversible cell cycle arrest involved in tumor suppression and aging. Many stresses, such as telomere shortening and oncogene activation, induce senescence by damaging nuclear DNA. However, the mechanisms linking DNA damage to senescence remain unclear. Here, we show that DNA damage response (DDR) signaling to mitochondria triggers senescence. A genome-wide small interfering RNA screen implicated the outer mitochondrial transmembrane protein BNIP3 in senescence induction. We found that BNIP3 is phosphorylated by the DDR kinase ataxia telangiectasia mutated (ATM) and contributes to an increase in the number of mitochondrial cristae. Stable isotope labeling metabolomics indicated that the increase in cristae enhances fatty acid oxidation (FAO) to acetyl–coenzyme A (acetyl-CoA). This promotes histone acetylation and expression of the cyclin-dependent kinase inhibitor p16^INK4a^. Notably, pharmacological activation of FAO alone induced senescence both in vitro and in vivo. Thus, mitochondrial energy metabolism plays a critical role in senescence induction and is a potential intervention target to control senescence.

## Teaser

Nuclear DNA damage activates fatty acid–fueled mitochondrial energy metabolism to induce cellular senescence, a hallmark of aging.

## INTRODUCTION

Cellular senescence is a stress-induced irreversible cell cycle arrest that is typically accompanied by the expression of the cyclin-dependent kinase (CDK) inhibitor p16^INK4a^ (hereafter referred to as p16) (*1*–*5*). Many senescence-inducing stresses, such as telomere shortening and oncogene activation, produce DNA damage, mainly double-strand breaks, and activate the DNA damage response (DDR) kinase ataxia telangiectasia mutated (ATM). ATM stabilizes and activates the transcription factor p53, which promotes the expression of the CDK inhibitor p21. p21 transiently arrests the cell cycle to provide time for DNA repair (*2*). If DNA damage persists, the resulting prolonged DDR signaling promotes the expression of p16, which maintains the state of cell cycle arrest (*1*–*3*). The genes encoding p53 (*TP53*) and p16 (*CDKN2A*) are frequently mutated or deleted in human cancers (*2*, *6*). Therefore, senescence is thought to be a tumor-suppressor mechanism that prevents the proliferation of potentially tumorigenic cells (*3*). In addition to CDK inhibitor expression, lysosomal β-galactosidase activity is high in senescent cells. The significance of this senescence-associated β-galactosidase (SA-β-gal) activity is unclear, but it is used in combination with others as a marker of senescence.

Recent studies have indicated that senescence contributes to aging and age-related diseases (*1*, *3*). p16 expression increases in various tissues with age (*7*, *8*). Increased p16 expression decreases the tissue-regenerative potential of pancreatic β cells and neuronal progenitor cells (*2*). Moreover, p16-expressing senescent cells that accumulate in the body impair heart and kidney function and shorten healthy lifespan (*9*). This impaired organ function may be due to the secretion of inflammatory cytokines and chemokines by senescent cells, a phenomenon termed the senescence-associated secretory phenotype (SASP) (*10*). The link between p16 and aging is further supported by the finding that the *INK4/ARF* locus, which encodes the senescence effectors p16, alternative reading frame (ARF), and p15, is a hotspot for susceptibility single-nucleotide polymorphisms associated with age-related diseases, such as atherosclerosis and diabetes (*1*). Therefore, elucidating the regulatory mechanisms of p16 expression may provide clues to understanding the mechanisms of aging. Numerous transcription factors and histone-modifying enzymes have been implicated in p16 expression (*6*). Notably, treatment of primary fibroblasts with histone deacetylase inhibitors has been reported to induce p16 expression and senescence-like proliferation arrest (*11*). However, little is known about the signaling pathways linking DNA damage to p16 expression (*3*).

Mitochondrial dysfunction is a feature of senescence (*1*, *12*, *13*). It has long been known that senescence is accompanied by an increase in mitochondrial reactive oxygen species (ROS) levels and a decrease in membrane potential (*14*). In addition, recent studies have reported senescence-associated changes in mitochondrial energy metabolism. Glucose and fatty acids are the primary fuels for mitochondrial energy metabolism and are oxidized to acetyl–coenzyme A (acetyl-CoA) in mitochondria (*15*). Acetyl-CoA enters the tricarboxylic acid (TCA) cycle, which produces the electron donors NADH {reduced form of NAD^+^ [nicotinamide adenine dinucleotide (oxidized form)]} and FADH_2_ to drive the respiratory chain for adenosine triphosphate (ATP) production. Levels of TCA cycle intermediates increase during senescence (*16*, *17*). In addition, respiratory activity has been shown to increase in a fatty acid oxidation (FAO)–dependent manner during oncogene-induced senescence (*18*). However, the mechanisms and significance of these mitochondrial metabolic changes remain poorly understood. Mitochondrial depletion through enforced mitophagy has been shown to prevent the development of senescence features, such as p16 expression, cell enlargement, SA-β-gal activity, and the SASP (*19*, *20*). Therefore, it seems certain that mitochondria are involved in the induction of senescence.

In this study, we performed a genome-wide small interfering RNA (siRNA) screen and found that BCL2/adenovirus E1B 19 kDa–interacting protein 3 (BNIP3), an outer mitochondrial transmembrane protein, is required for DNA damage–induced p16 expression. Mass spectrometric analysis of BNIP3-interacting proteins yielded ATM and subunits of the mitochondrial contact site and cristae organizing system (MICOS) complex. We found that upon DNA damage, BNIP3 is phosphorylated by ATM and contributes to an increase in the number of mitochondrial cristae. This increase in cristae enhances the oxidation of fatty acids to acetyl-CoA, an acetyl group donor, thereby promoting histone acetylation around the *p16* transcription start site (TSS). Notably, pharmacological activation of FAO alone can induce senescence in fibroblasts, endothelial cells, and mouse liver. Our findings identify BNIP3 as a key molecule in the mitochondrial DDR and reveal the importance of FAO activation by BNIP3 in p16 expression and senescence induction.

## RESULTS

### BNIP3 links DNA damage to p16 expression

Treatment with doxorubicin, which produces DNA double-strand breaks, increased p16 expression in IMR-90 primary human fibroblasts (fig. S1, A to D). Consistent with previous studies (*7*, *21*), this increase was suppressed by ATM knockdown but was enhanced by p53 knockdown. These results suggest that downstream effectors of ATM other than p53 mediate DNA damage–induced p16 expression. Since the effect of ATM knockdown on p16 expression was partial (fig. S1, A to C), ATM-independent pathways may also contribute to p16 expression.

**Fig. 1.**
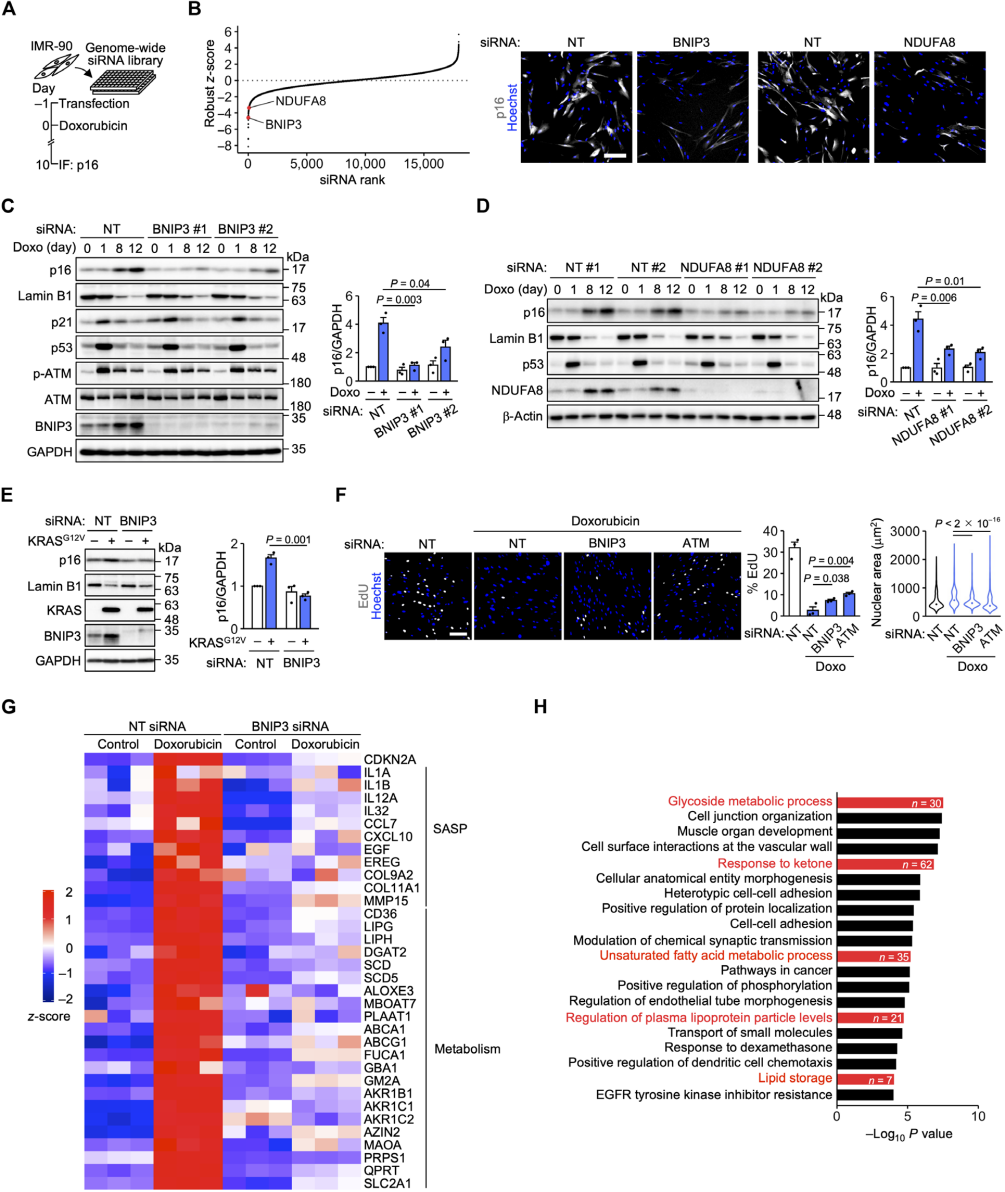
BNIP3 links DNA damage to p16 expression. (**A**) Schematic of the siRNA screen. IF, immunofluorescence. (**B**) Left, robust *z*-scores of the screened siRNAs. Right, images of IMR-90 cells transfected with nontargeting (NT), BNIP3, and NDUFA8 siRNAs from the siRNA screen. p16 is shown in gray. Scale bar, 200 μm. (**C** and **D**) Immunoblot analysis of IMR-90 cells transfected with the indicated siRNAs and treated with doxorubicin (Doxo). *n* = 3 independent experiments. (**E**) Immunoblot analysis of IMR-90 cells expressing ER-KRAS^G12V^ for 10 days. *n* = 3 independent experiments. (**F**) Left, EdU assay using IMR-90 cells treated with Doxo (50 ng/ml) for 24 hours and incubated in fresh medium for 7 days. EdU is shown in gray. Scale bar, 200 μm. Center, percentage of EdU-positive cells. *n* = 3 biological replicates. Right, distribution of the nuclear area. Dot, median. *n* = 3215 (siNT), 5487 (siNT + Doxo), 4802 (siBNIP3 + Doxo), and 7857 (siATM + Doxo) cells. Data are means ± SEM. (**G**) Heatmap of expression levels of SASP genes and metabolism-related genes in IMR-90 cells transfected with BNIP3 #1 siRNA and treated with Doxo for 12 days. (**H**) GO analysis of genes whose expression is increased by Doxo treatment in a BNIP3-dependent manner. Statistical analysis was performed using Dunnett’s multiple comparison test [C, D, and F (center)], unpaired two-tailed Student’s *t* test (E), and the Wilcoxon rank-sum test with Bonferroni correction [F (right)].

By combining anti-p16 immunofluorescence and automated microscopy, we established a high-throughput p16 quantification assay that can be used in large-scale screens (fig. S1E) (*22*). The specificity of the p16 antibody was confirmed with a p16 siRNA (fig. S1F). We used this assay to carry out a genome-wide siRNA screen for genes required for DNA damage–induced p16 expression (Fig. 1A). IMR-90 cells were transfected with siRNAs in 384-well plates. p16 expression was induced by doxorubicin treatment and quantified on day 10. Genes whose knockdown significantly suppressed p16 expression included *BNIP3*, which encodes an outer mitochondrial transmembrane protein previously implicated in cell death and mitophagy (*23*), and *NDUFA8*, which encodes an essential subunit of respiratory chain complex I (Fig. 1B and table S1) (*24*). Also included were *SMARCB1*, *SMARCA4*, and *PRBM1*, which encode components of the SWI/SNF complex, a chromatin remodeler known to promote p16 expression (*6*).

We confirmed that knockdown of BNIP3 or NDUFA8 suppressed doxorubicin-induced p16 expression in IMR-90 cells and human umbilical vein endothelial cells (HUVECs) (Fig. 1, C and D, and fig. S2, A to D). BNIP3 knockdown also suppressed oncogenic RAS (ER-KRAS^G12V^)–induced p16 expression (Fig. 1E). Doxorubicin treatment increased BNIP3 and NDUFA8 levels along with p16 levels (Fig. 1, C and D, and fig. S2, C and D). BNIP3 knockdown only slightly attenuated the loss of lamin B1, a senescence marker (*25*), and did not markedly affect the activation of the ATM-p53-p21 pathway (Fig. 1C). Neither BNIP3 nor NDUFA8 knockdown decreased the number of γH2AX foci, a marker of DNA damage (fig. S2E). In a mouse mammary tumor model, BNIP3 has been found to prevent tumor cell proliferation and tumor growth (*26*). We examined whether BNIP3 is involved in senescence-associated proliferation arrest. Doxorubicin treatment decreased the percentage of 5-ethynyl-2′-deoxyuridine (EdU)–positive proliferating cells and concomitantly increased nuclear size (Fig. 1F). These changes were attenuated by BNIP3 or ATM knockdown.

We performed RNA sequencing (RNA-seq) to investigate the involvement of BNIP3 in senescence-associated transcriptomic changes. In addition to p16, BNIP3 was involved in the expression of a subset of SASP factors, such as interleukin-1α (IL-1α) and IL-1β, during doxorubicin-induced senescence (Fig. 1G). Using quantitative polymerase chain reaction (qPCR), we confirmed that BNIP3 knockdown suppressed the expression of IL-1α and IL-1β during both doxorubicin- and oncogenic RAS–induced senescence (fig. S2, F and G). Gene ontology (GO) analysis revealed that BNIP3 was also involved in the expression of metabolism-related genes, such as glycoside and lipid metabolism–related genes, during doxorubicin-induced senescence (Fig. 1, G and H).

BNIP3 has been reported to promote mitochondrial ROS production and loss of mitochondrial membrane potential during cell death (*23*). BNIP3 knockdown suppressed the increase in mitochondrial ROS levels and the decrease in mitochondrial membrane potential during doxorubicin-induced senescence (fig. S2, H and I). BNIP3 knockdown decreased ATP levels in doxorubicin-induced senescent cells (fig. S2J). Although overexpression of p16 has been reported to result in SA-β-gal activity (*27*), knockdown of p16, BNIP3, or NDUFA8 did not significantly affect the percentage of SA-β-gal–positive cells (fig. S2K), suggesting that p16 is not essential for SA-β-gal activity. These results indicate that BNIP3 is involved in p16 expression and several other features of senescence.

### BNIP3 is a mitochondrial substrate of ATM

**Fig. 2.**
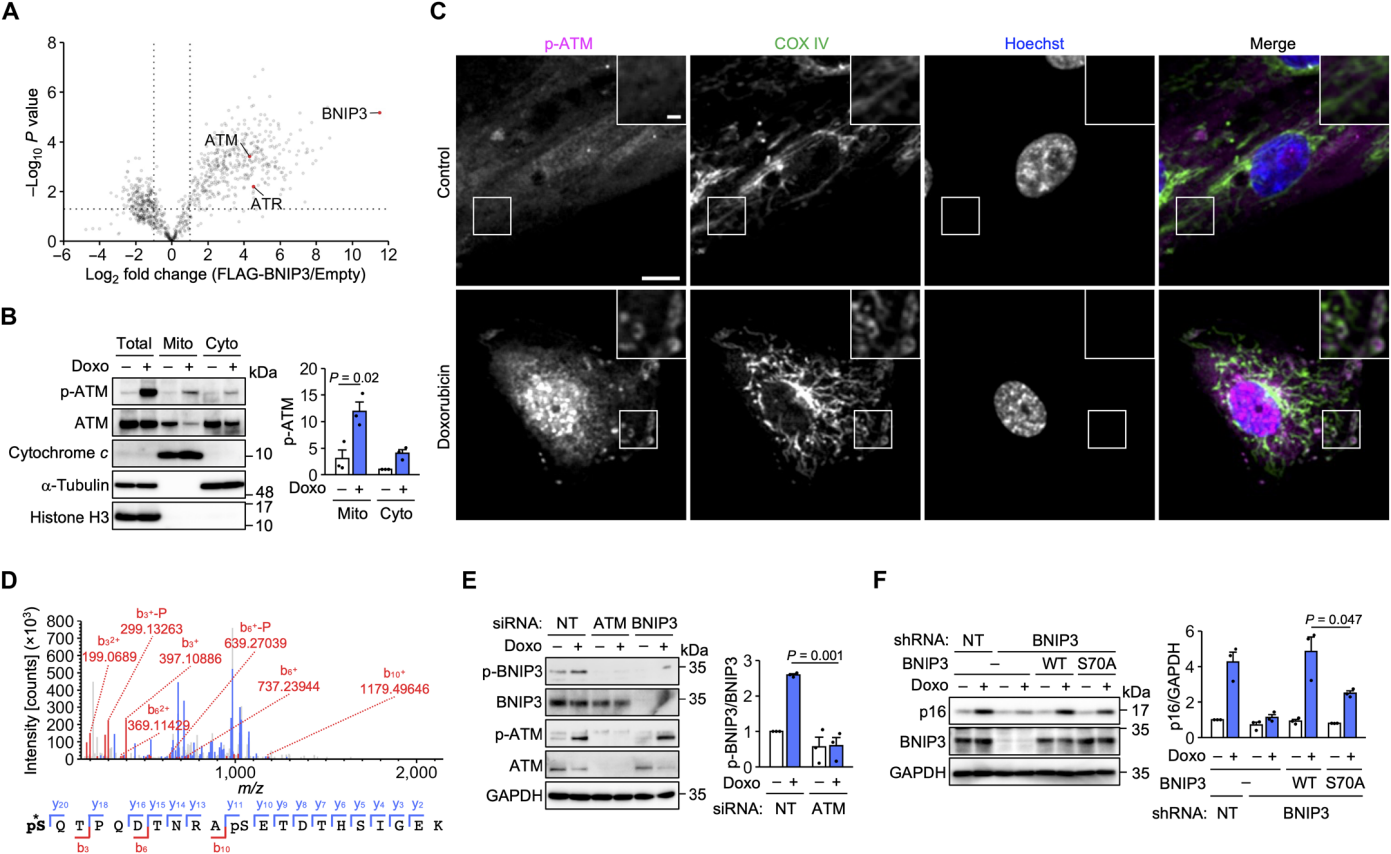
BNIP3 is a mitochondrial substrate of ATM. (**A**) Volcano plot of mass spectrometric identification of FLAG-BNIP3–interacting proteins. *n* = 3 biological replicates. (**B**) Subcellular fractionation of IMR-90 cells treated with doxorubicin (Doxo) for 24 hours. The same amount of protein was loaded in each lane. *n* = 3 independent experiments. (**C**) Immunofluorescence analysis of IMR-90 cells treated with Doxo for 24 hours. Magenta, p-ATM; green, COX IV (mitochondrial marker). Scale bar, 10 μm; inset scale bar, 2 μm. (**D**) Top, product ion spectrum of a BNIP3-derived peptide containing phosphorylated S70. Bottom, identified b and y ions. The asterisk denotes S70. (**E**) Immunoblot analysis of IMR-90 cells transfected with NT, ATM, and BNIP3 #1 siRNAs and treated with Doxo for 24 hours. *n* = 3 independent experiments. (**F**) Immunoblot analysis of IMR-90 cells infected with the indicated lentiviruses and treated with Doxo (100 ng/ml) for 12 days. *n* = 3 independent experiments. Data are means ± SEM. Statistical analysis was performed using unpaired two-tailed Student’s *t* test.

BNIP3 is a transmembrane protein of the outer mitochondrial membrane (*23*). We confirmed that BNIP3 was localized to mitochondria in both control and doxorubicin-treated cells (fig. S3, A and B). The mechanisms by which DNA damage affects mitochondrial function during senescence remain elusive (*12*). p53 can regulate mitochondrial proteins (*28*), but it was dispensable for p16 expression (fig. S1D). To investigate the regulation and function of BNIP3, we identified BNIP3-interacting proteins by mass spectrometry. FLAG-BNIP3 was overexpressed in human embryonic kidney (HEK) 293T cells and immunoprecipitated with anti-FLAG beads. The coprecipitated proteins included ATM and ATM- and Rad3-related (ATR) (Fig. 2A and table S2), a DDR kinase that is activated by single-stranded DNA and shares many substrates with ATM (*29*).

ATM is localized in part to mitochondria and contributes to the maintenance of respiratory activity (*30*), possibly by phosphorylating unidentified mitochondrial substrates (*29*). Subcellular fractionation of IMR-90 cells showed the presence of ATM in the mitochondrial fraction (Fig. 2B). Notably, doxorubicin treatment for 24 hours increased phosphorylated ATM levels in the mitochondrial and cytosolic fractions. Immunofluorescence analysis showed that phosphorylated ATM was localized mainly to the nucleus but also to a subset of mitochondria (Fig. 2C). These results suggest that a portion of ATM activated in the nucleus translocates to mitochondria.

We examined the possibility that BNIP3 is a mitochondrial substrate of ATM. The substrate motifs of ATM and ATR are both SQ/TQ (*29*). A phospho-SQ antibody recognized BNIP3 overexpressed in HEK293T cells (fig. S4A). Mutational analysis indicated that of the three candidate residues, S70 was the phosphorylation site. Phosphorylation at this site was also detected by mass spectrometry (Fig. 2D). S70 and its surrounding sequences are conserved among species (fig. S4B). We raised a phospho-specific antibody against S70 of BNIP3. As expected, this antibody recognized immunoprecipitated FLAG-BNIP3 in an S70-dependent manner (fig. S4C). Coprecipitation of ATM was also confirmed. We then investigated the phosphorylation state of endogenous BNIP3. Treatment with doxorubicin increased the phosphorylation of BNIP3 at S70 from day 1 in IMR-90 cells (Fig. 2E and fig. S4D). This increase was suppressed by ATM knockdown (Fig. 2E). In addition, intraperitoneal injection of doxorubicin in mice increased the levels of phosphorylated ATM and phosphorylated BNIP3 in the mitochondrial fraction of the liver (fig. S4E). Furthermore, ATM phosphorylated BNIP3 in vitro (fig. S4F). To examine the significance of BNIP3 phosphorylation, BNIP3^WT^ and BNIP3^S70A^ were reexpressed in BNIP3 knockdown cells. Doxorubicin-induced p16 expression was restored almost fully by BNIP3^WT^ but only partially by BNIP3^S70A^ (Fig. 2F). These results suggest that BNIP3 is an ATM substrate that mediates DNA damage–induced p16 expression.

### BNIP3 increases the number of cristae

**Fig. 3.**
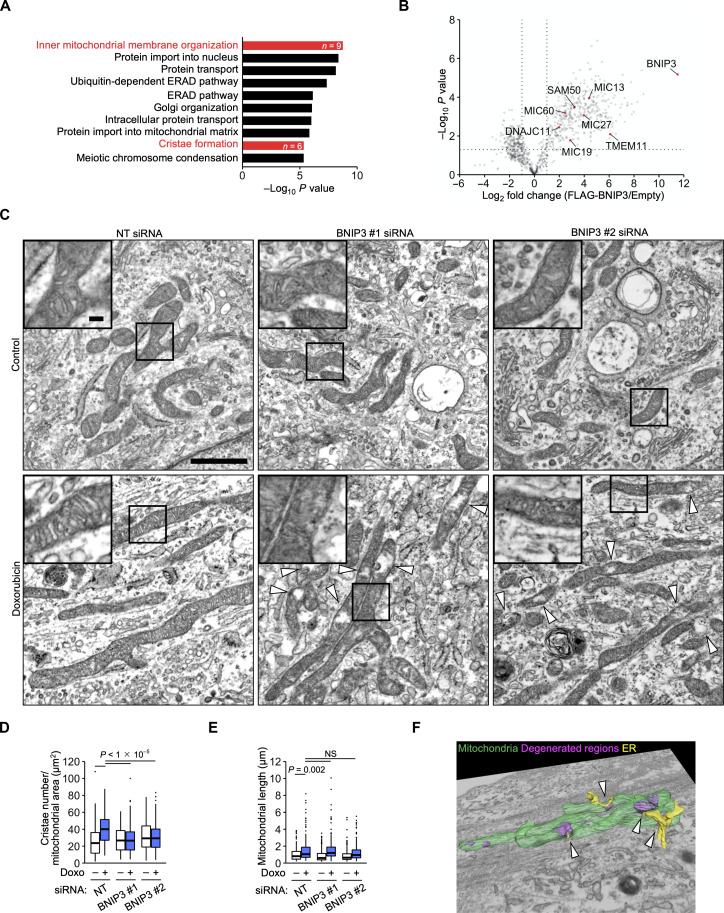
BNIP3 increases the number of cristae. (**A**) GO analysis of BNIP3-interacting proteins identified by mass spectrometry. (**B**) Volcano plot of mass spectrometric identification of FLAG-BNIP3–interacting proteins. *n* = 3 biological replicates. (**C**) Electron microscopy of IMR-90 cells transfected with NT and BNIP3 siRNAs and treated with doxorubicin (Doxo) for 12 days. The arrowheads denote degenerated regions. Scale bar, 1 μm; inset scale bar, 100 nm. (**D** and **E**) Quantification of the number of cristae per mitochondrial area (D) and mitochondrial length (E). *n* = 217 (siNT), 149 (siNT + Doxo), 286 (siBNIP3 #1), 173 (siBNIP3 #1 + Doxo), 168 (siBNIP3 #2), and 203 (siBNIP3 #2 + Doxo) mitochondria. Center line, median; box limits, upper and lower quartiles; whiskers, 1.5× interquartile range; points, outliers. Statistical analysis was performed using the Wilcoxon rank-sum test with Bonferroni correction. NS, not significant. (**F**) FIB-SEM of IMR-90 cells transfected with BNIP3 #1 siRNA and treated with Doxo for 12 days. Green, mitochondria; magenta, degenerated regions; yellow, ER. The arrowheads denote mitochondria-ER contact sites.

BNIP3 was initially thought to be a proapoptotic BCL2 family protein when it was found (*23*). More recently, it has been regarded as a mitophagy receptor that interacts with LC3 family proteins (*26*). However, mitophagy is impaired during senescence, perhaps through mitochondrial elongation (*12*). Inhibition of autophagic degradation with bafilomycin A1 did not significantly affect BNIP3 levels in senescent cells (fig. S5A). In contrast, treatment with MG-132, a proteasome inhibitor, increased BNIP3 levels. To explore BNIP3 function, we performed GO analysis of the BNIP3-interacting proteins. This analysis revealed the most significant enrichment for “inner mitochondrial membrane organization” proteins, including the MICOS complex subunits MIC60, MIC19, MIC13, and MIC27 and their interacting proteins TMEM11, SAM50, and DNAJC11 (Fig. 3, A and B, and table S2). MICOS is an inner mitochondrial membrane complex with dual functions: formation of contact sites with the outer membrane and maintenance of the architecture of inner membrane invaginations known as cristae (*31*). Although a recent study identified BNIP3 as a TMEM11-interacting protein (*32*), the relationship between BNIP3 and MICOS has not been tested. We confirmed that MIC60 coprecipitated with FLAG-BNIP3 by immunoblotting (fig. S5B). The interaction between FLAG-BNIP3 and MIC60 was slightly increased by doxorubicin treatment. This increase was abolished by the S70A mutation. MIC60 is a core subunit whose loss destabilizes the other subunits and MICOS-interacting proteins (*31*). MIC60 knockdown decreased BNIP3 levels along with MIC19 levels in IMR-90 cells (fig. S5C). In contrast, BNIP3 knockdown did not markedly affect MIC60 or MIC19 levels (fig. S5D).

We examined whether BNIP3 alters mitochondrial structure and dynamics during DNA damage–induced senescence. Using electron microscopy, we found that doxorubicin treatment increased the number of cristae per mitochondrial area in IMR-90 cells (Fig. 3, C and D). This increase was suppressed by BNIP3 knockdown with partial degeneration of mitochondria. Focused ion beam scanning electron microscopy (FIB-SEM) revealed that the degenerated regions tended to be adjacent to endoplasmic reticulum (ER)–mitochondria contact sites (Fig. 3F), where MICOS has been proposed to facilitate lipid trafficking (*31*). Doxorubicin treatment also induced mitochondrial elongation (Fig. 3, C and E), as reported in replicative senescence (*12*). This elongation was not affected by BNIP3 knockdown. NDUFA8 knockdown did not suppress the increase in cristae number or mitochondrial length (fig. S6, A to C). As in IMR-90 cells, doxorubicin treatment increased the number of cristae in HUVECs (fig. S6, D to F). This was also suppressed by BNIP3 knockdown. Next, we examined mitochondrial dynamics using live-cell imaging. In control IMR-90 cells, mitochondria were confirmed to undergo cycles of fission and fusion (movie S1). In doxorubicin-induced senescent cells, mitochondria formed reticular networks that were also continuously reorganized by fission and fusion (movie S2). Mitochondrial dynamics did not appear to be affected by BNIP3 knockdown (fig. S6G and movies S3 and S4). These results suggest that BNIP3 is a MICOS-interacting protein that increases the number of cristae during DNA damage–induced senescence.

### BNIP3 activates mitochondrial FAO

**Fig. 4.**
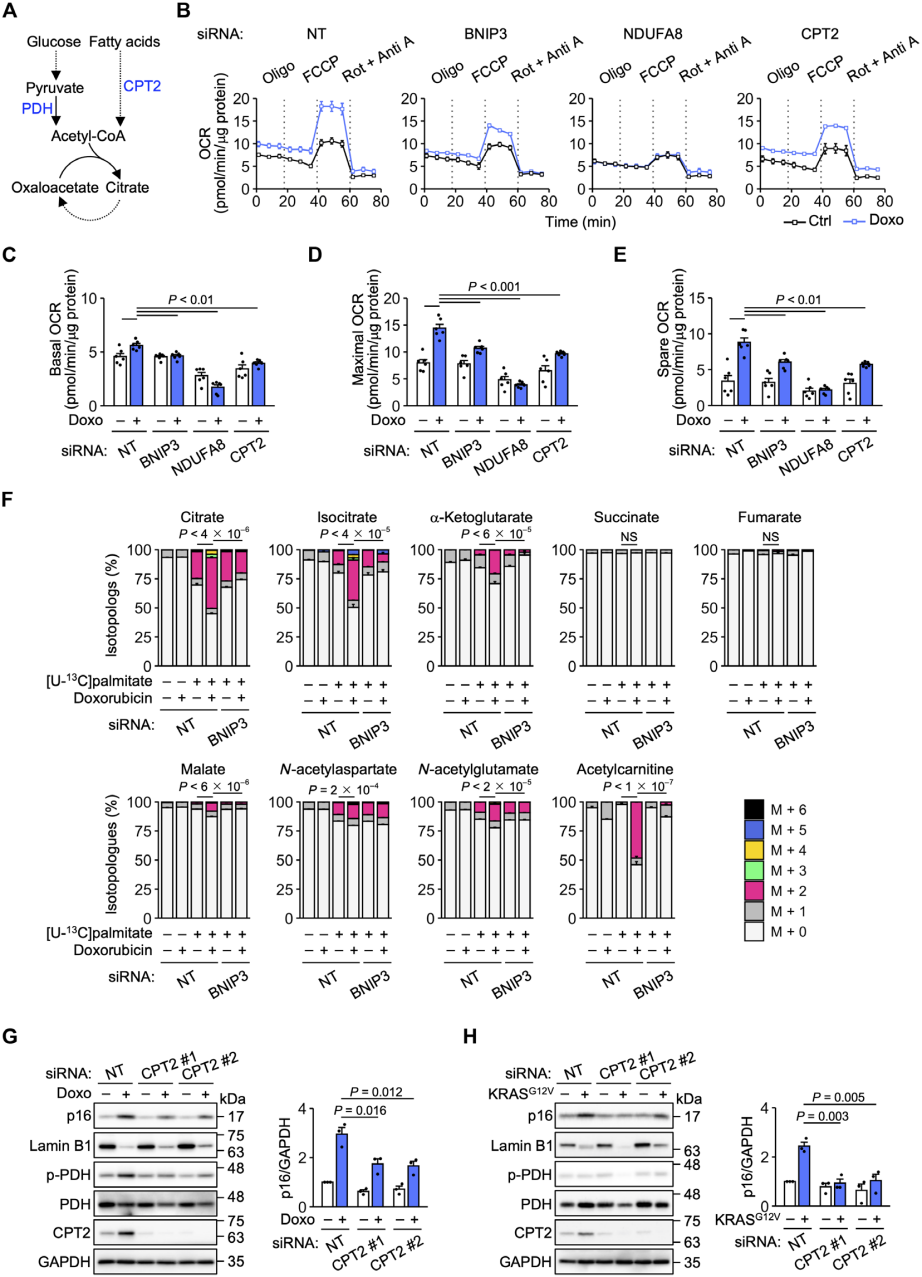
BNIP3 activates mitochondrial FAO. (**A**) Schematic of acetyl-CoA and the TCA cycle. (**B**) Oxygen consumption rate (OCR) measurements in IMR-90 cells transfected with NT, BNIP3 #1, NDUFA8 #1, and CPT2 #1 siRNAs and treated with doxorubicin (Doxo) for 12 days. *n* = 6 biological replicates. (**C** to **E**) Basal OCR (C), maximal OCR (D), and spare OCR (E) were calculated from (B). (**F**) Distributions of the isotopologs containing ^13^C derived from labeled palmitate in control and doxorubicin-induced senescent IMR-90 cells transfected with the indicated siRNAs. (**G** and **H**) Immunoblot analysis of IMR-90 cells transfected with NT and CPT2 siRNAs and treated with Doxo (G) or expressing ER-KRAS^G12V^ (H) for 12 days. *n* = 3 independent experiments. Statistical analysis was performed using Dunnett’s multiple comparison test. NS, not significant.

Mitochondrial cristae are the sites of oxidative phosphorylation and house respiratory chain complexes and ATP synthase (*31*). Respiratory chain complex I, which oxidizes NADH to NAD^+^ (*33*), was implicated in p16 expression by our siRNA screen (Fig. 1B). In addition to its role in membrane potential generation, the supply of NAD^+^ by complex I is essential for mitochondrial metabolic pathways such as the TCA cycle and FAO (*15*, *33*). Metabolites of these pathways act as signaling molecules to regulate gene expression (*33*). We reasoned that BNIP3 promotes p16 expression by altering mitochondrial metabolism. Metabolomic analysis showed that doxorubicin treatment significantly increased the levels of the TCA cycle intermediates citrate, isocitrate, and α-ketoglutarate without increasing the levels of pyruvate (fig. S7A), a glycolytic product that enters the TCA cycle via conversion to acetyl-CoA (Fig. 4A). Doxorubicin treatment also increased the levels of other acetyl-CoA–derived metabolites such as acetylcarnitine, hexosamine pathway metabolites, and acetylated amino acids, but not β-hydroxybutyrate (fig. S7A). These increases tended to be suppressed by knockdown of BNIP3, ATM, or NDUFA8, raising the possibility that acetyl-CoA metabolism is involved in p16 expression.

FAO is a metabolic pathway in which acetyl-CoA is produced from the breakdown of fatty acids (*15*). FAO activation during differentiation and starvation involves mitochondrial elongation and an increase in the number of cristae (*34*–*37*). Doxorubicin treatment increased mitochondrial oxygen consumption (Fig. 4, B to E). This increase was suppressed by knockdown of BNIP3, NDUFA8, or carnitine palmitoyltransferase 2 (CPT2), a mitochondrial enzyme involved in long-chain FAO (*15*). To evaluate FAO activity, we performed isotope labeling with [U-^13^C]palmitate. [U-^13^C]palmitate-derived carbons were incorporated into TCA intermediates, acetylated amino acids, and acetylcarnitine more efficiently in senescent IMR-90 cells and HUVECs than in proliferating cells (Fig. 4F and fig. S7B). The difference in the rate of incorporation into acetylcarnitine was particularly evident. In contrast, the difference in the rate of incorporation into *N*-acetylaspartate appears to be small, but it should be noted that *N*-acetylaspartate levels are markedly higher in senescent cells (fig. S7A). The increase in the incorporation of [U-^13^C]palmitate-derived carbons was suppressed by BNIP3 knockdown (Fig. 4F and fig. S7B). The incorporation of [U-^13^C]palmitate-derived carbons into acetyl-CoA also increased in senescent IMR-90 cells (fig. S7C). Fluorescent probe analysis confirmed that doxorubicin treatment increased FAO activity (fig. S8A). In addition, doxorubicin treatment decreased the size of lipid droplets (fig. S8B), storage organelles for fatty acids and sterols (*37*). This increase in FAO activity and decrease in lipid droplet size was suppressed by knockdown of BNIP3, NDUFA8, or CPT2 (fig. S8, A to D). Furthermore, doxorubicin- or oncogenic RAS–induced p16 expression was attenuated by CPT2 knockdown (Fig. 4, G and H). These results suggest that FAO activation by BNIP3 promotes p16 expression. We noticed that doxorubicin treatment and oncogenic RAS expression increased CPT2 levels (Fig. 4, G and H). This increase may also contribute to FAO activation.

FAO activation decreases acetyl-CoA production from glucose (*38*). This effect, known as the Randle cycle, is primarily due to inhibitory phosphorylation of pyruvate dehydrogenase (PDH), the mitochondrial enzyme that converts pyruvate to acetyl-CoA (Fig. 4A). Isotope labeling with [U-^13^C]glucose indicated that the conversion of pyruvate to acetyl-CoA and then to citrate and acetylated amino acids decreased during doxorubicin-induced senescence (fig. S9A). In addition, doxorubicin treatment increased PDH phosphorylation (Fig. 4G and fig. S9, B to D). This increase was attenuated by the knockdown of CPT2, BNIP3, or ATM but not by knockdown of p16. In contrast to doxorubicin treatment, oncogenic RAS expression did not increase PDH phosphorylation (Fig. 4H), perhaps reflecting that PDH phosphorylation is not solely regulated by FAO (*39*). These results are consistent with FAO activation during DNA damage–induced senescence.

### Pharmacological activation of FAO induces senescence

**Fig. 5.**
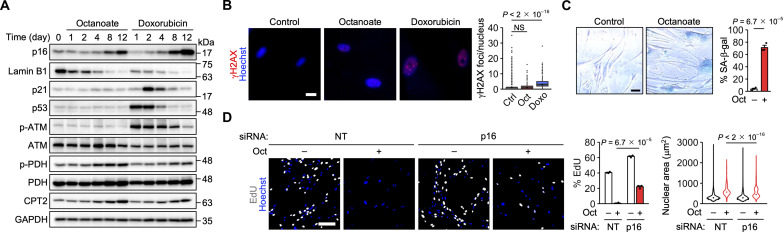
FAO activation with the medium-chain fatty acid octanoate induces senescence. (**A**) Immunoblot analysis of IMR-90 cells treated with octanoate (Oct) or doxorubicin (Doxo). (**B**) Left, immunofluorescence analysis of IMR-90 cells treated with 10 mM Oct and Doxo (100 ng/ml) for 12 days. γH2AX is shown in red. Scale bar, 20 μm. Right, distribution of the number of γH2AX foci. Center line, median; box limits, upper and lower quartiles; whiskers, 1.5× interquartile range; points, outliers. *n* = 3588 (Ctrl), 471 (Oct), and 792 (Doxo) cells. (**C**) SA-β-gal staining in IMR-90 cells treated with Oct for 12 days. Scale bar, 50 μm. *n* = 3 biological replicates. (**D**) Left, EdU assay using IMR-90 cells transfected with NT and p16 siRNAs and treated with Oct for 12 days. EdU is shown in gray. Scale bar, 200 μm. Center, percentage of EdU-positive cells. *n* = 3 biological replicates. Right, distribution of the nuclear area. Dot, median. *n* = 6766 (siNT), 1005 (siNT + Oct), 9551 (sip16), and 2832 (sip16 + Oct) cells. Data are means ± SEM. Statistical analysis was performed using the Wilcoxon rank-sum test with Bonferroni correction [B and D (right)] and unpaired two-tailed Student’s *t* test [C and D (center)]. NS, not significant.

**Fig. 6.**
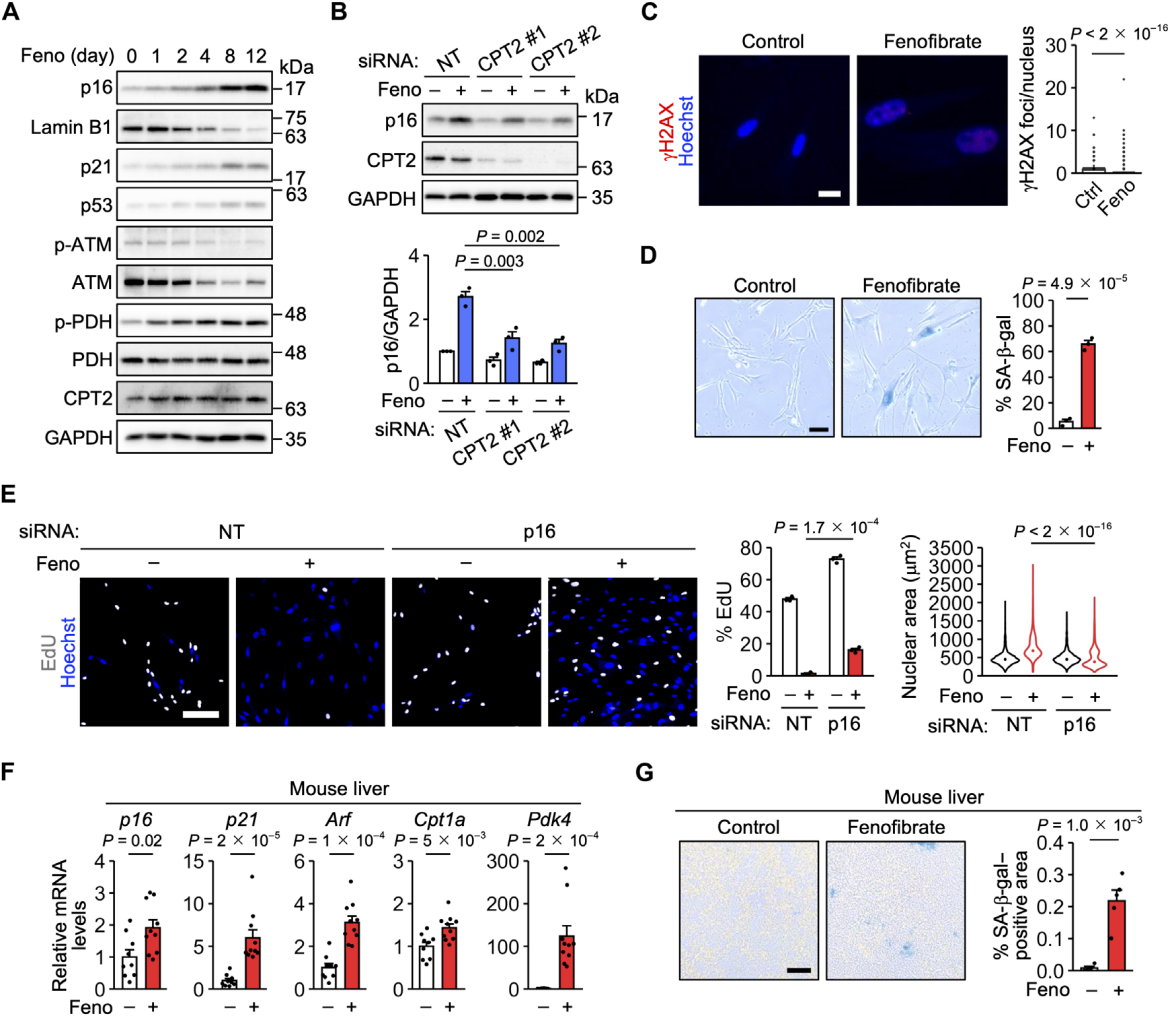
FAO activation with the PPARα agonist fenofibrate induces senescence. (**A**) Immunoblot analysis of IMR-90 cells treated with fenofibrate (Feno). (**B**) Immunoblot analysis of IMR-90 cells transfected with NT and CPT2 siRNAs and treated with Feno for 12 days. *n* = 3 independent experiments. (**C**) Left, immunofluorescence analysis of IMR-90 cells treated with Feno for 12 days. γH2AX is shown in red. Scale bar, 20 μm. Right, distribution of the number of γH2AX foci. *n* = 1694 (Ctrl) and 1292 (Feno) cells. (**D**) SA-β-gal staining in IMR-90 cells treated with Feno for 12 days. Scale bar, 50 μm. *n* = 3 biological replicates. (**E**) Left, EdU assay using IMR-90 cells transfected with NT and p16 siRNAs and treated with Feno for 8 days. EdU is shown in gray. Scale bar, 200 μm. Center, percentage of EdU-positive cells. *n* = 3 biological replicates. Right, distribution of the nuclear area. *n* = 1907 (siNT), 3370 (siNT + Feno), 2119 (sip16), and 9776 (sip16 + Feno) cells. (**F**) qPCR analysis of livers from 8- or 10-week-old mice treated with Feno for 3 weeks. *n* = 9 (Ctrl) and 10 (Feno) mice. (**G**) SA-β-gal staining in livers from 10-week-old mice treated with Feno for 3 weeks. *n* = 4 (Ctrl) and 5 (Feno) mice. Data are means ± SEM. Statistical analysis was performed using Dunnett’s multiple comparison test (B), the Wilcoxon rank-sum test with Bonferroni correction [C and E (right)], and unpaired two-tailed Student’s *t* test [D, E (center), F, and G].

To further investigate the importance of FAO in senescence induction, we used the medium-chain fatty acid octanoate. Unlike the more abundant long-chain fatty acids, octanoate freely crosses mitochondrial membranes (*40*). When added to cells, octanoate is readily oxidized to acetyl-CoA, an acetyl group donor, and promotes histone acetylation and the associated expression of lipid metabolism–related genes. Treatment of IMR-90 cells with octanoate increased p16, CPT2, and phosphorylated PDH levels and decreased lamin B1 levels (Fig. 5A). In contrast, the ATM-p53-p21 pathway and γH2AX foci were largely unaffected (Fig. 5, A and B). Octanoate treatment increased the percentage of SA-β-gal–positive cells (Fig. 5C). In addition, octanoate treatment decreased the percentage of EdU-positive proliferating cells and increased nuclear size (Fig. 5D and fig. S10A). These changes in cell proliferation and nuclear size were suppressed by p16 knockdown (Fig. 5D). Octanoate treatment increased the expression of inflammatory cytokines and chemokines (fig. S10B). Similar results were obtained with fenofibrate, a lipid-lowering drug that activates FAO through the transcription factor peroxisome proliferator–activated receptor α (PPARα) (Fig. 6, A to E, and fig. S10, C and D) (*41*). We confirmed that fenofibrate-induced p16 expression was attenuated by CPT2 knockdown (Fig. 6B). Treatment with octanoate or fenofibrate increased mitochondrial ROS levels, decreased mitochondrial membrane potential, and increased the number of cristae and mitochondrial length (fig. S10, E to I).

BNIP3 knockdown suppressed octanoate- and fenofibrate-induced p16 expression and proliferation arrest (fig. S11, A, C, D, and F), suggesting that BNIP3 can be involved in FAO even in the absence of DNA damage. NDUFA8 knockdown yielded similar results (fig. S11, B, C, E, and F), as expected since complex I is essential for FAO (*15*).

As in IMR-90 cells, treatment with octanoate or fenofibrate resulted in senescence features, including p16 expression, in TIG-3 primary human fibroblasts and HUVECs (fig. S12, A to I). Furthermore, treatment of mice with fenofibrate for 3 weeks increased the expression of p16, p21, and ARF in the liver, along with the expression of the PPARα transcriptional targets CPT1A and pyruvate dehydrogenase kinase 4 (PDK4) (Fig. 6F). ARF is a p53 activator encoded by an alternative reading frame of *CDKN2A*, which also encodes p16, and is thought to be important for mouse senescence (*2*). Fenofibrate treatment also increased SA-β-gal staining in the liver (Fig. 6G). These results suggest that pharmacological activation of FAO alone can induce senescence without activating DDR signaling.

### Histone acetylation around the *p16* TSS increases during senescence

**Fig. 7.**
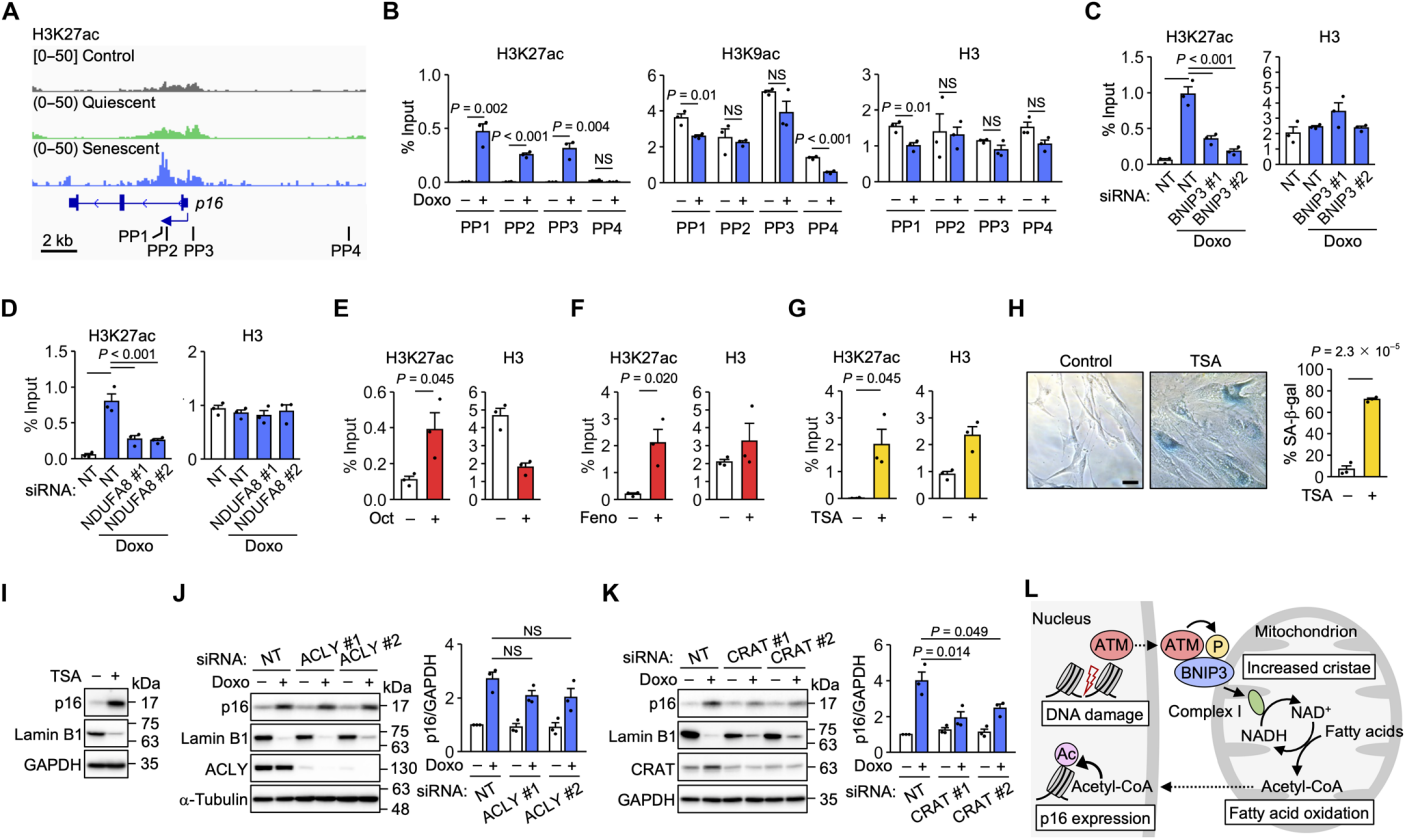
Histone acetylation around the *p16* TSS increases during senescence. (**A**) H3K27ac ChIP-seq data analysis of HRAS^G12V^-induced senescent cells (GSE74328). (**B**) ChIP-qPCR analysis of IMR-90 cells treated with doxorubicin (Doxo) for 12 days using primer pairs (PPs) targeting the regions indicated in (A). *n* = 3 independent experiments. (**C** and **D**) ChIP-qPCR analysis of IMR-90 cells transfected with the indicated siRNAs and Doxo for 12 days using PP1. (**E** to **G**) ChIP-qPCR analysis of IMR-90 cells treated with octanoate (Oct) for 14 days (E), fenofibrate (Feno) for 12 days (F), or TSA for 12 days (G) using PP1. *n* = 3 independent experiments. (**H**) SA-β-gal staining in IMR-90 cells treated with TSA for 12 days. Scale bar, 50 μm. *n* = 3 biological replicates. (**I**) Immunoblot analysis of IMR-90 cells treated with TSA for 12 days. (**J** and **K**) Immunoblot analysis of IMR-90 cells transfected with the indicated siRNAs and treated with Doxo. *n* = 3 independent experiments. (**L**) Model for the mitochondrial response to nuclear DNA damage in senescence induction. Data are means ± SEM. Statistical analysis was performed using unpaired two-tailed Student’s *t* test (B, E, F, G, and H) and Dunnett’s multiple comparison test (C, D, J, and K). NS, not significant.

To examine the possibility that FAO activation by BNIP3 promotes p16 expression through histone acetylation, we analyzed publicly available chromatin immunoprecipitation sequencing (ChIP-seq) data. This analysis revealed that acetylation of histone H3 at lysine 27 (H3K27ac) increased around the *p16* TSS during oncogenic RAS–induced senescence (Fig. 7A). In general, increased H3K27ac is associated with transcriptional activation (*42*). ChIP-qPCR analysis indicated that doxorubicin treatment also increased H3K27ac, but not H3K9ac, around the *p16* TSS (Fig. 7B). This increase was suppressed by the knockdown of BNIP3 or NDUFA8 (Fig. 7, C and D). Treatment with octanoate or fenofibrate also increased H3K27ac (Fig. 7, E and F). Furthermore, treatment with the histone deacetylase inhibitor trichostatin A (TSA) increased H3K27ac and p16 levels, decreased lamin B1 levels, and conferred SA-β-gal activity (Fig. 7, G to I).

To supply the nucleus with acetyl-CoA for histone acetylation, acetyl-CoA produced in the mitochondria is transported out of the mitochondria, primarily via conversion to citrate (*33*). Citrate is converted back to acetyl-CoA by ATP citrate lyase (ACLY) in the cytosol and nucleus. However, knockdown of ACLY did not appear to affect doxorubicin-induced p16 expression (Fig. 7J). In addition to citrate, acetylcarnitine has recently been reported to mediate the transport of acetyl-CoA (*43*). Doxorubicin treatment increased the levels of carnitine acetyltransferase (CRAT) (Fig. 7K), which produces acetylcarnitine. Knockdown of CRAT attenuated p16 expression. These results raise the possibility that FAO activation increases p16 expression via the nuclear supply of acetyl-CoA for histone acetylation.

## DISCUSSION

In this study, we have shown that nuclear DNA damage signaling to mitochondria via BNIP3 induces senescence by activating FAO (Fig. 7L). Our results suggest that DNA damage–activated ATM translocates to mitochondria, where it phosphorylates BNIP3. BNIP3 increases the number of cristae and activates FAO. This activation may reflect an attempt to repair DNA damage through histone acetylation (*44*). If DNA damage persists, the resulting prolonged FAO activation appears to promote histone acetylation for p16 expression. However, histones are not the only proteins whose acetylation state is regulated by acetyl-CoA levels (*45*). In addition, FAO activation has effects other than increased acetyl-CoA levels (*15*, *38*, *41*). In particular, increased mitochondrial ROS production may contribute to the induction of senescence (*12*, *46*). Further studies are needed to elucidate the mechanisms by which BNIP3 regulates cristae and by which FAO drives senescence. We used doxorubicin and oncogenic RAS, which cause DNA damage (*1*), to conclude that DNA damage–activated FAO drives senescence. However, we cannot completely exclude the possibility that doxorubicin activates FAO independently of DNA damage.

To understand the role of BNIP3 in senescence in vivo, a study using BNIP3 knockout mice would be warranted. Previous studies have reported that BNIP3 knockout mice exhibit increased tumorigenesis in breast and lung cancer models and are resistant to the cardiotoxicity of doxorubicin (*26*, *47*, *48*). Given the role of senescence in tumor suppression and doxorubicin cardiotoxicity (*3*, *49*), the reported phenotypes of BNIP3 knockout mice may be due to impaired senescence induction.

BNIP3 has been reported to attenuate age-related muscle inflammation (*50*). This is due to the preservation of mitochondrial and lysosomal function but may also be due in part to the induction of senescence. Senescent cells are generally thought to promote age-related chronic inflammation via the SASP (*1*, *51*). However, given recent evidence that senescent cells play a role in maintaining tissue structure and function (*52*), their presence may contribute to the prevention of chronic inflammation in certain tissues.

Using RNA-seq, we found that BNIP3 increased the expression of glycoside and lipid metabolism–related genes during senescence. The mechanism of this increase is unclear, but FAO activation with octanoate has been reported to increase the expression of lipid metabolism–related genes via histone acetylation (*40*). Therefore, the increased expression of lipid metabolism–related genes by BNIP3 may also be due to FAO activation.

Tumorigenesis requires metabolic changes to meet the bioenergetic, biosynthetic, and redox demands of increased cell proliferation (*53*). In some breast cancers and B cell lymphomas, FAO is activated by oncogene activation or overexpression of fatty acid–metabolizing enzymes (*41*). FAO is also activated by a high-fat diet and promotes the cancerous transformation of intestinal stem cells (*54*). Furthermore, ovarian cancer metastasizes to the omentum, an organ composed primarily of adipocytes, presumably because the oxidation of fatty acids supplied by adipocytes supports tumor growth (*55*). Mechanistically, FAO is thought to promote cancer cell proliferation and survival through the production of NADPH, a metabolite critical for biosynthesis and redox regulation (*41*). We speculate that FAO-induced senescence may have evolved to counteract FAO-dependent tumorigenesis and that other metabolic changes involved in tumorigenesis may also induce senescence.

In contrast to our results in DNA damage–induced senescence, a previous study reported that oncogenic RAF-induced senescence involves PDH dephosphorylation (*56*). This difference may suggest that prolonged activation of acetyl-CoA production can induce senescence regardless of the carbon source, depending on the context.

Senescent cells accumulate in the body over time and contribute to aging (*1*). However, the stresses that cause this accumulation are still unknown. FAO is activated in response to fasting, exercise, and intake of a high-fat diet (*15*, *54*). Medium-chain fatty acids are produced primarily from triglycerides carried in milk and dairy products, while short-chain fatty acids are produced when gut bacteria digest dietary fiber (*57*). A high-fat diet and butyrate, a short-chain fatty acid, have been reported as senescence-inducing stimuli (*58*, *59*), but it is not known whether they require FAO to induce senescence. Aging is thought to be accompanied by a shift in fuel preference toward fatty acids (*60*). We speculate that prolonged FAO activation, whether due to DNA damage or not, may contribute to the accumulation of senescent cells with age.

DNA damage has been proposed to be the primary cause of aging, in part because most premature aging syndromes result from mutations in DNA repair genes (*61*). Of note, these syndromes and normal aging are accompanied by mitochondrial dysfunction (*61*, *62*). However, the mechanisms of this mitochondrial dysfunction remain poorly understood. The known mechanisms by which DNA damage affects mitochondria are largely limited to the regulation of mitochondrial biogenesis and mitophagy via transcription factors (*63*, *64*). A recent study in DNA repair–deficient *Caenorhabditis elegans* showed that DNA damage shortens the lifespan through FAO activation and histone hyperacetylation (*65*). Another study showed that the accumulation of histone modifications associated with DNA repair contributes to aging in mice (*66*). Moreover, cristae morphology was found to change with age in mice and *Drosophila* (*67*). Further analysis of the mitochondrial response to nuclear DNA damage may shed light on the mechanisms of aging and lead to the identification of targets for antiaging interventions.

## MATERIALS AND METHODS

### Cell culture and treatments

IMR-90 primary human fibroblasts [American Type Culture Collection (ATCC), CCL-186], TIG-3 primary human fibroblasts (Japanese Cancer Research Resources Bank, JCRB0506), and HEK293T cells (ATCC, CRL-3216) were cultured in Dulbecco’s modified Eagle’s medium (DMEM, Sigma-Aldrich, D5796; Wako, 044-29765) supplemented with 10% fetal bovine serum (BioWest, S1560-500). IMR-90 cells and TIG-3 cells were used at population doubling level 40 or lower. HUVECs (Lonza, C2517A) were cultured in EGM-2 BulletKit medium (Lonza, CC-3162). Unless otherwise indicated, the cells were treated with doxorubicin hydrochloride (200 ng/ml; Wako, 040-21521), 5 mM sodium octanoate (Tokyo Chemical Industry, O0034, adjusted to pH 7.4 with HCl), 50 μM fenofibrate (Abcam, ab120832), or 0.5 μM TSA (Wako, 209-17563). MG-132 (Enzo Life Sciences, BML-PI102-0005) and bafilomycin A1 (Cayman Chemical, 11038) were added to final concentrations of 10 and 1 μM, respectively. To induce ER-KRAS^G12V^ expression, the cells were treated with 200 nM 4-hydroxytamoxifen (Cayman Chemical, 17308).

### Plasmids

To produce lentiviruses encoding short hairpin RNAs (shRNAs), shRNA sequences containing the following target sequences were cloned into the pLKO.1-TRC cloning vector (Addgene, 10878): nontargeting (NT), 5′-CAACAAGATGAAGAGCACCAA-3′; ATM #1, 5′-CCTGCCAACATACTTTAAGTA-3′; ATM #2, 5′-CGAGATCCTGAAACAATTAAA-3′; p53 #1, 5′-GACTCCAGTGGTAATCTAC-3′; p53 #2, 5′-GAGGGATGTTTGGGAGATGTA-3′; and BNIP3, 5′-GCTTCTGAAACAGATACCCAT-3′. To construct BNIP3 expression vectors, human BNIP3 was cloned into the pENTR or pENTR-5′-FLAG entry vector. Site-directed mutagenesis was performed to replace S2, S70, and S93 with alanine. To construct pENTR-shRNA–resistant BNIP3, site-directed mutagenesis was performed with the following primers: 5′-GATACCAACAGAGCAAGCGAAACAGATACCC-3′ and 5′-GGGTATCTGTTTCGCTTGCTCTGTTGGTATC-3′. LR reactions were performed using a Gateway LR Clonase enzyme mix (Invitrogen, 11791-100) and the pcDNA3-DEST or pLenti-PGK-Hygro (Addgene, 19066) destination vector. pLenti-PGK-ER-KRAS^G12V^ was purchased from Addgene (35635).

### Lentivirus infection

For lentivirus production, HEK293T cells were transfected with a lentiviral vector and the packaging plasmids pCMV-VSV-G (Addgene, 8454) and psPAX2 (Addgene, 12260) using PEI MAX transfection reagent (Polyscience, 24765) for 18 hours. The medium was replaced with 5 ml of fresh medium containing 1% bovine serum albumin (BSA, Iwai Chemical, A001) and collected 24 hours later. This step was repeated. The collected media were combined and filtered through a 0.45-μm pore size filter (Millipore, SLHVR33RS). IMR-90 cells were infected with lentiviruses in the presence of polybrene (8 μg/ml; Nacalai Tesque, 17736-44) for 18 hours, cultured in fresh medium for 24 hours, and selected with puromycin (0.5 to 1.0 μg/ml; Gibco, A11138-03) or hygromycin (50 to 100 μg/ml; Nacalai Tesque, 07296-11) for at least 2 days.

### Immunoblotting and IP

Cells were lysed in radioimmunoprecipitation assay (RIPA) buffer [50 mM tris-HCl (pH 8.0), 150 mM NaCl, 1% NP-40, 0.5% sodium deoxycholate, 0.1% SDS, 1 mM phenylmethylsulfonyl fluoride, leupeptin (5 μg/ml), 8 mM NaF, 12 mM β-glycerophosphate, 1 mM Na_3_VO_4_, 1.2 mM Na_2_MoO_4_, 5 μM cantharidin, and 2 mM imidazole] or IP lysis buffer [20 mM tris-HCl (pH 7.5), 150 mM NaCl, 10 mM EDTA (pH 8.0), 1% Triton X-100, 1 mM phenylmethylsulfonyl fluoride, leupeptin (5 μg/ml), 8 mM NaF, 12 mM β-glycerophosphate, 1 mM Na_3_VO_4_, 1.2 mM Na_2_MoO_4_, 5 μM cantharidin, and 2 mM imidazole]. Mitochondria were isolated using a mitochondria isolation kit (Qiagen, 37612) according to the manufacturer’s protocol and lysed in RIPA buffer. The lysates were clarified by centrifugation at 13,000*g* for 15 min at 4°C. The protein concentrations of the lysates were quantified using a bicinchoninic acid (BCA) protein assay kit (Wako, 297-73101) and equalized. The lysates were then mixed with 2× SDS sample buffer [125 mM tris-HCl (pH 6.8), 4% SDS, 20% glycerol, bromophenol blue (200 μg/ml), 10% β-mercaptoethanol] and heated at 98°C for 3 min. IP was performed with anti-FLAG M2 beads (Sigma-Aldrich, A2220). The beads were washed three times with IP lysis buffer and heated in 2× SDS sample buffer. The SDS samples were subjected to SDS–polyacrylamide gel electrophoresis (SDS-PAGE) and transferred to Immobilon-P membranes (Millipore, IPVH00010). The membranes were blocked with 5% skim milk (Yukijirushi) in TBS-T [50 mM tris-HCl (pH 8.0), 150 mM NaCl, and 0.05% Tween 20] for 1 hour at room temperature and incubated with primary antibodies in TBS-T containing 5% BSA and 0.1% NaN_3_ overnight at 4°C. The membranes were then incubated with secondary antibodies in TBS-T containing 5% skim milk for 30 min at room temperature. ECL Select detection reagent (Amersham, RPN2235) was used for detection on a FUSION Solo S chemiluminescence imaging system (Vilber). Blots were quantified using ImageJ software (National Institutes of Health). The following primary antibodies were used: anti-p16 (Abcam, ab108349), anti-ATM (Abcam, ab32420), anti–α-tubulin (Bio-Rad, MCA77G), anti–phospho-ATM S1981 (Abcam, ab81292), anti-p21 (Abcam, ab109199), anti-p53 (Santa Cruz Biotechnology, sc-126), anti–lamin B1 (Abcam, ab16048), anti-BNIP3 (Cell Signaling Technology, 44060), anti–glyceraldehyde-3-phosphate dehydrogenase (GAPDH, Proteintech, 60004-1-Ig), anti-NDUFA8 (Abcam, ab184952), anti–β-actin (Sigma-Aldrich, A3853), anti-KRAS (Santa Cruz Biotechnology, sc-30), anti–cytochrome *c* (BD Biosciences, 556433), anti–histone H3 (Abcam, ab1791), anti–phospho-ATM/ATR substrate (SQ) (Cell Signaling Technology, 9607), anti-BNIP3 (Abcam, ab109362), anti–phospho-ATM S1981 (Active Motif, 39530), anti-CPT2 (Santa Cruz Biotechnology, sc-377294), anti–β-catenin (Santa Cruz Biotechnology, sc-7963), anti-p62 (Santa Cruz Biotechnology, sc-28359), anti-MIC60 (Proteintech, 10179-1-AP), anti-MIC19 (Proteintech, 25625-1-AP), anti–PDH E1α (Santa Cruz Biotechnology, sc-377092), anti–phospho-PDH E1α S293 (Abcam, ab177461), anti-ACLY (Abcam, ab40793), and anti-CRAT (Proteintech, 15170-1-AP). A phospho-specific antibody against BNIP3 S70 was raised by immunizing a rabbit with a keyhole limpet hemocyanin-conjugated phosphopeptide (CDSPPR[pS]QTPQD) and affinity-purified (Eurofins).

### Quantitative PCR

Total RNA was extracted using ISOGEN (Wako, 319-90211) and subjected to reverse transcription using a ReverTra Ace master mix (Toyobo, FSQ-301). qPCR was performed using a SYBR FAST qPCR kit (KAPA Biosystems, KK4602) and a QuantStudio 1 qPCR system (Applied Biosystems). The following primers were used: *GAPDH* (human): forward, 5′-AGCCACATCGCTCAGACAC-3′, reverse, 5′-GCCCAATACGACCAAATCC-3′; *p16* (human): forward, 5′-CCAACGCACCGAATAGTTACG-3′, reverse, 5′-GCGCTGCCCATCATCATG-3′; *BNIP3* (human): forward, 5′-TGCTGCTCTCTCATTTGCTG-3′, reverse, 5′-GACTCCAGTTCTTCATCAAAAGGT-3′; *NDUFA8* (human): forward, 5′-ATGCCGGGGATAGTGGAG-3′, reverse, 5′-GCACAGCAGAACTAATTTTCACC-3′; *IL1A* (human): forward, 5′-GCCAGCCAGAGAGGGAGTC-3′, reverse, 5′-TGGAACTTTGGCCATCTTGAC-3′; *IL1B* (human): forward, 5′-TACCTGTCCTGCGTGTTGAA-3′, reverse, 5′-TCTTTGGGTAATTTTTGGGATCT-3′; *IL6* (human): forward, 5′-CCGGGAACGAAAGAGAAGCT-3′, reverse, 5′-GCGCTTGTGGAGAAGGAGTT-3′; *CCL2* (human): forward, 5′-AGTCTCTGCCGCCCTTCT-3′, reverse, 5′-GTGACTGGGGCATTGATTG-3′; *IL8* (human): forward, 5′-CTTTCCACCCCAAATTTATCAAAG-3′, reverse, 5′-CAGACAGAGCTCTCTTCCATCAGA-3′; *Rps18* (mouse): forward, 5′-TCCAGCACATTTTGCGAGTA-3′, reverse, 5′-CAGTGATGGCGAAGGCTATT-3′; *p16* (mouse): forward, 5′-CCCAACGCCCCGAACT-3′, reverse, 5′-GCAGAAGAGCTGCTACGTGAA-3′; *Arf* (mouse): forward, 5′-GCTCTGGCTTTCGTGAACATG-3′, reverse, 5′-TCGAATCTGCACCGTAGTTGAG-3′; *p21* (mouse): forward, 5′-CTGGTGATGTCCGACCTGTT-3′, reverse, 5′-TCAAAGTTCCACCGTTCTCG-3′; *Cpt1a* (mouse): forward, 5′-CTCCGCCTGAGCCATGAAG-3′, reverse, 5′-CACCAGTGATGATGCCATTCT-3′; and *Pdk4* (mouse): forward, 5′-CCGCTTAGTGAACACTCCTTC-3′, reverse, 5′-TGACCAGCGTGTCTACAAACT-3′. The transcript levels were analyzed using the ΔΔ*C*_t_ method with *GAPDH* (human) or *Rps18* (mouse) as an internal control.

### siRNA transfection

siRNAs with the following target sequences were purchased from Dharmacon: NT (siGENOME), 5′-AUGAACGUGAAUUGCUCAA-3′; ATM (siGENOME), 5′-GCAAAGCCCUAGUAACAUA-3′; p16 (siGENOME), 5′-AAACUUAGAUCAUCAGUCA-3′; NT #1 (ON-TARGETplus), 5′-UGGUUUACAUGUCGACUAA-3′; NT #2 (ON-TARGETplus), 5′-UGGUUUACAUGUUGUGUGA-3′; BNIP3 #1 (ON-TARGETplus), 5′-GGAAAGAAGUUGAAAGCAU-3′; BNIP3 #2 (ON-TARGETplus), 5′-GGAAUUAAGUCUCCGAUUA-3′; NDUFA8 #1 (ON-TARGETplus), 5′-GAAAACAGAUCGACCUUUA-3′; NDUFA8 #2 (ON-TARGETplus), 5′-GUAGAUGAGGUGAAAAUUA-3′; ATM (ON-TARGETplus), 5′-GCAAAGCCCUAGUAACAUA-3′; MIC60 #1 (ON-TARGETplus), 5′-GGGAUGACUUUAAACGAGA-3′; MIC60 #2 (ON-TARGETplus), 5′-CAGACAAACUCUUCGAGAU-3′; CPT2 #1 (ON-TARGETplus), 5′-CUAGAUGACUUCCCCAUUA-3′;CPT2 #2 (ON-TARGETplus), 5′-GGGCCUACCUGGUCAAUGC-3′; p16 (ON-TARGETplus), 5′-GAUCAUCAGUCACCGAAGG-3′; ACLY #1 (ON-TARGETplus), 5′-GCACGAAGUCACAAUCUUU-3′; ACLY #2 (ON-TARGETplus), 5′-CGAGUGAAGUCGAUAAACA-3′; CRAT #1 (ON-TARGETplus), 5′-GUACCACAGUGACGGGACA-3′; and CRAT #2 (ON-TARGETplus), 5′-CCAAGAAGCUGCGGUUCAA-3′. ON-TARGETplus and the #1 siRNAs were used unless otherwise indicated. siRNA transfection was performed at a final concentration of 10 nM using Lipofectamine RNAiMAX transfection reagent (Invitrogen, 13778500) and Opti-MEM (Gibco, 31985070) for 24 to 48 hours.

### Genome-wide siRNA screening

siGENOME SMARTpool siRNA libraries (human drug targets, G-004655-E2; human druggable subsets, G-004675-E2; human genome, G-005005-E2) were purchased from Dharmacon. The siRNA in each well was diluted to 375 nM in siRNA buffer (Dharmacon, B-002000-UB-100). A total of 1.5 pmol of siRNA (4 μl) was dispensed into each well of black clear-bottom 384-well plates (Greiner, 781091) with a Biomek FX^P^ liquid handler (Beckman Coulter). Lipofectamine RNAiMAX transfection reagent and Opti-MEM were mixed at a volume ratio of 3:400 and added to each well using a Multidrop Combi automated pipetting machine (Thermo Fisher Scientific). The cells were transfected with siRNAs at a final concentration of 30 nM for 24 hours and treated with doxorubicin (250 ng/ml) for 10 days. For immunofluorescence, the cells were fixed with 4% paraformaldehyde in phosphate-buffered saline (PBS) for 10 min and permeabilized with 0.1% Triton X-100 in PBS containing 1% BSA for 5 min. The cells were washed with PBS using an AquaMax microplate washer (Molecular Devices) and incubated with an anti-p16 antibody (BD Biosciences, 551154, 1:1000) in PBS containing 1% BSA overnight at 4°C. After washing with PBS, the cells were incubated with Alexa Fluor Plus 647 (Invitrogen, A32728, 1:1000) and Hoechst 33258 (Dojindo, H341, 1:2000) in PBS containing 1% BSA for 1 hour at room temperature and washed with PBS. Images were acquired with a CellInsight NXT automated microscope (Thermo Fisher Scientific) and analyzed with HCS Studio software (Thermo Fisher Scientific). The mean fluorescence intensity of p16 in the nucleus was quantified and log-transformed to achieve a near-normal distribution. The robust *z*-score of each siRNA was calculated. siRNAs with cell counts below 30 were excluded from the analysis.

### Immunofluorescence

To count γH2AX foci, cells were fixed with 4% paraformaldehyde in PBS for 10 min and permeabilized with 0.1% Triton X-100 in PBS containing 1% BSA for 5 min. The cells were incubated with anti-γH2AX (Abcam, ab2893) antibody in PBS containing 1% BSA for 1 hour and then with Alexa Fluor secondary antibodies (Invitrogen, 1:1000) and Hoechst 33258 (1:2000) for 1 hour. Images were acquired with a CellInsight NXT automated microscope and analyzed with HCS Studio software. For confocal microscopy, the cells were grown on glass coverslips (Matsunami Glass, C015001), fixed with 4% paraformaldehyde in PBS for 10 min, and permeabilized with 0.1% Triton X-100 in PBS containing 1% BSA for 5 min. The cells were incubated with anti-BNIP3 (Abcam, ab109362), anti-TOM20 (Santa Cruz Biotechnology, sc-17764), anti–phospho-ATM S1981 (Active Motif, 39530), and COX IV (Proteintech, 11242-1-AP) antibodies in PBS containing 1% BSA for 1 hour and then with Alexa Fluor secondary antibodies (Invitrogen, 1:1000) and Hoechst 33258 (1:2000) for 1 hour. Images were acquired using a TCS SP5 confocal microscope (Leica) with a 63× oil immersion objective [numerical aperture (NA) 1.40, Leica].

### EdU assay

A Click-iT EdU imaging kit (Invitrogen, C10340) was used. The cells were incubated with 10 μM EdU for 24 hours, fixed with 4% paraformaldehyde in PBS for 15 min at room temperature, and washed twice with PBS containing 3% BSA. The cells were permeabilized with 0.5% Triton X-100 in PBS for 20 min at room temperature and washed with PBS containing 3% BSA. Click-iT reaction cocktail was added and allowed to react for 30 min at room temperature. After washing with PBS containing 3% BSA, the cells were incubated with Hoechst 33342 (1:2000) in PBS for 30 min at room temperature. Images were acquired with a CellInsight NXT automated microscope and analyzed with HCS Studio software.

### RNA-seq

Total RNA was extracted using ISOGEN. Three biological replicates were performed for each condition. Libraries were prepared and sequenced by Azenta. Sequence reads were mapped to the human reference genome GRCh38 using Hisat2 (v.2.2.1). Transcript read counts were determined using featureCounts (v.2.0.6). Differential gene expression analysis was performed using the R (v.4.3.2) package Deseq2 (v.1.42.1). Genes with adjusted *P* value <0.1 and fold change >2 (NT siRNA control versus NT siRNA doxorubicin) and <1/1.5 (NT siRNA doxorubicin versus BNIP3 siRNA doxorubicin) were subjected to GO analysis using Metascape.

### ROS measurement

Cells were incubated with 5 μM MitoSOX mitochondrial superoxide indicator (Invitrogen, M36008) and Hoechst 33342 (Dojindo, H342, 1:1000) in serum-free DMEM for 30 min at 37°C and washed with PBS. To evaluate autofluorescence, the cells were also stained with Hoechst 33342 only. Images were acquired with a CellInsight NXT automated microscope and analyzed with HCS Studio software.

### Measurement of mitochondrial membrane potential

Cells were incubated with 5 μM JC-1 (Cayman Chemical, 15003) in culture medium for 15 min at 37°C and washed twice with Hanks’ balanced salt solution (HBSS; 137 mM NaCl, 5.4 mM KCl, 4.2 mM NaHCO_3_, 0.34 mM Na_2_HPO_4_, 0.44 mM KH_2_PO_4_, and 5.6 mM glucose pH 7.4). Fluorescence was measured with a Varioskan microplate reader (Ex/Em = 485/535 nm and Ex/Em = 535/595 nm).

### SA-β-gal staining

SA-β-gal staining was performed as described elsewhere (*68*). The cells were washed twice with PBS, fixed with 4% paraformaldehyde in PBS for 3 min, and washed twice with PBS. The cells were incubated in SA-β-gal staining solution [X-gal (1 mg/ml; Tokyo Chemical Industry, B3201), 40 mM citric acid/sodium phosphate buffer (pH 6.0), 5 mM potassium ferricyanide, 5 mM potassium ferrocyanide, 150 mM NaCl, and 2 mM MgCl_2_] for 48 hours at 37°C.

C57BL/6J male mice were fed CE-2 powdered chow (CLEA Japan) with or without fenofibrate (0.5% w/w) for 3 weeks, euthanized, and perfused with PBS. Livers were harvested, embedded in Cryomount I (Muto Pure Chemicals, 33351), and frozen in liquid nitrogen. Sections were cut at 5-μm thickness using a Leica CM3050S cryostat (Leica) at −20°C, mounted on MAS-coated glass slides (Matsunami Glass, MAS-04), fixed with 1% paraformaldehyde in PBS for 1 min, washed three times with PBS, and immersed in SA-β-gal staining solution for 48 hours at 37°C.

### Protein mass spectrometry

HEK293T cells were transfected with pcDNA3 empty vector and pcDNA3-FLAG-BNIP3 in 10-cm dishes for 24 hours, lysed in IP lysis buffer, and centrifuged at 15,000*g* for 15 min at 4°C. The supernatants were incubated with FLAG M2 beads for 30 min at 4°C. The beads were washed with IP lysis buffer and incubated with 3× FLAG peptide (5 mg/ml; Sigma-Aldrich, F4799; M&S TechnoSystems, GEN-3-FLAG-5) in TBS [10 mM tris-HCl (pH 7.4) and 150 mM NaCl] for 30 min at 4°C. After centrifugation, the supernatants were collected in low-protein–binding tubes (Eppendorf, 0030108442). Trichloroacetic acid was added to the supernatants to a final concentration of 10%. The supernatants were incubated on ice for 30 min and centrifuged at 15,000*g* for 10 min at 4°C. The precipitates were resuspended in 200 μl of ice-cold acetone and centrifuged at 15,000*g* for 10 min at 4°C. The precipitates were then resuspended in ultrapure water (Wako, 214-01301) containing 50 mM ammonium bicarbonate and 0.1% RapiGest surfactant (Waters, 186002123) and heated for 10 min at 98°C. The samples were shaken for 5 min and subjected to ultrasonication. The samples were then treated with 5 mM tris(2-carboxyethyl)phosphine for 10 min at 98°C, 10 mM methyl methanethiosulfonate for 30 min at room temperature, and 1 μg of mass spectrometry–grade trypsin (Promega, V5280) overnight at 37°C. Trifluoroacetic acid was added to reach 0.5%. The samples were incubated for 40 min at 37°C and centrifuged at 20,000*g* for 10 min at 4°C. The supernatants were desalted using a GL-Tip SDB kit (GL Sciences, 7820-11200) according to the manufacturer’s protocol, except that the columns were washed twice with 200 μl of solution A. For phosphorylation analysis, phosphopeptides were enriched using a Titansphere Phos-TiO kit (GL Sciences, 5010-21305) before desalting. After vacuum centrifugation, the peptides were dissolved in 0.1% formic acid for protein identification or in 0.1% formic acid and 2% acetonitrile for phosphorylation analysis, clarified by centrifugation at 20,000*g* for 10 min at room temperature, and transferred to ultralow-adsorption vials (AMR, PSVial100).

Liquid chromatography tandem mass spectrometry (LC-MS/MS) analysis was performed on a Q Exactive mass spectrometer (Thermo Fisher Scientific) connected to an EASY-nLC 1200 system (Thermo Fisher Scientific). Peptides were loaded onto a C_18_ trap column (Thermo Fisher Scientific, 164946) and a C_18_ analytical column (Nikkyo Technos, NTCC-360/75-3-123) and separated at a flow rate of 300 nl/min with a gradient of mobile phase A (0.1% formic acid in ultrapure water) and mobile phase B (0.1% formic acid in acetonitrile) according to the following protocols: 0 to 10% B for 5 min, 10 to 40% B for 85 min, 40 to 95% B for 2 min, and 95% B for 18 min for protein identification; and 2 to 40% B for 85 min, 40 to 95% B for 5 min, 95% B for 10 min, 95 to 2% B for 2 min, and 2% B for 8 min for phosphorylation analysis. The following MS settings were used: range, 400 to 1,500 mass/charge ratio (*m/z*) (protein identification) or 350 to 1500 *m/z* (phosphorylation analysis); resolution, 70,000; automatic gain control target, 3E6; and maximum injection time, 60 ms. The following MS^2^ settings were used: topN, 10; normalized collision energy, 28 (protein identification) or 27 (phosphorylation analysis); isolation window, 3.0 *m/z* (protein identification) or 1.6 *m/z* (phosphorylation analysis); resolution, 17,500; automatic gain control target, 1E5 (protein identification) or 5E5 (phosphorylation analysis); and maximum injection time, 60 ms (protein identification) or 100 ms (phosphorylation analysis). The data were analyzed with Proteome Discoverer software (Thermo Fisher Scientific). GO analysis was performed using DAVID Bioinformatics Resources.

### In vitro kinase assay

FLAG-BNIP3 overexpressed in HEK293T cells was purified by IP and incubated with recombinant ATM (Sigma-Aldrich, 14-933) in kinase buffer [20 mM Hepes (pH 7.5), 50 mM NaCl, 10 mM MgCl_2_, 10 mM MnCl_2_, 1 mM dithiothreitol, and 5 μM ATP] for 20 min at 30°C.

### Electron microscopy

Cells were fixed with 2% paraformaldehyde and 2% glutaraldehyde in 0.1 M phosphate buffer (pH 7.4) overnight at 4°C and postfixed with 2% osmium tetroxide for 2 hours at 4°C. The cells were washed in distilled water for 10 min three times and block-stained with 1% uranyl acetate for 30 min. The cells were then washed in distilled water three times, dehydrated with a graded series of ethanol, and embedded in Epon 812 resin (Oken Shoji) for 72 hours at 60°C. Ultrathin sections (100 nm) were cut with a Leica EM UC6 ultramicrotome (Leica), placed on 12-mm circular glass coverslips, and stained with uranyl acetate and lead citrate. The coverslips were mounted on aluminum pin stubs with carbon tape (Nissin EM) and carbon paste (Pelco Colloidal Graphite) and coated using a carbon coater (CADE-4 T, Meiwafosis) to prevent electron charging. Electron micrographs were acquired using a Helios Nanolab 660 FIB–SEM (Thermo Fisher Scientific) equipped with a backscattered electron detector (circular backscatter detector) at an acceleration voltage of 2.0 kV and a current of 0.4 nA. Mitochondrial area and length were quantified using the “freehand” tool of ImageJ software. Cristae were counted manually.

### Focused ion beam scanning electron microscopy

Resin-embedded samples were glued to aluminum pin stubs, cut with a Leica EM UC6 ultramicrotome, and coated with a thin layer of Pt-Pd using an MC1000 ion sputter coater (Hitachi High Technologies). Serial electron micrographs were acquired every 10 nm using a Helios Nanolab 660 FIB-SEM equipped with a backscattered electron detector (MD detector) at an acceleration voltage of 2.0 kV and a current of 0.4 nA. FIB milling was performed at an acceleration voltage of 30 kV with a beam current of 0.4 nA. Three-dimensional reconstruction was performed using Amira software (Thermo Fisher Scientific).

### Live-cell imaging of mitochondria

The cells were grown in 35-mm glass-bottom dishes (Matsunami Glass, D11130H), incubated with 200 nM MitoTracker Green FM (Invitrogen, M7514) in HBSS for 30 min at 37°C, and washed with HBSS. Images were acquired every 15 s at 37°C using a TCS SP5 confocal microscope (Leica) with a 63× oil immersion objective (NA 1.40, Leica) and a stage-top incubator (Tokai Hit).

### Metabolomic analysis

The cells were grown in 10-cm dishes, washed twice with ice-cold PBS, and lysed in 1 ml of methanol containing 25 μM l-methionine sulfone and 25 μM MES as internal standards and 400 μl of ultrapure water. The lysate was mixed with 800 μl of chloroform and centrifuged at 10,000*g* for 3 min at 4°C. The aqueous layer was transferred to a centrifugal filter (Human Metabolome Technologies, UFC3LCCNB-HMT) and centrifuged at 9100*g* for 3 hours at 4°C. The solvent of the filtrate was evaporated by blowing nitrogen gas for 2 hours at 40°C (TAITEC). The residue was dissolved in 25 μl of ultrapure water.

The levels of anionic metabolites were quantified using a Q Exactive Focus mass spectrometer (Thermo Fisher Scientific) connected to an ICS-5000+ chromatography compartment (Thermo Fisher Scientific). The samples were separated on a Dionex IonPac AS11-HC column (4-μm particle size, Thermo Fisher Scientific) at a flow rate of 0.25 ml/min with a gradient of potassium hydroxide according to the following protocol: 1 to 100 mM (0 to 40 min), 100 mM (40 to 50 min), and 1 mM (50 to 60 min). The column temperature was set at 30°C. A post-column make-up flow of methanol was added at a flow rate of 0.18 ml/min. A Dionex AERS 500 anion electrolytic suppressor (Thermo Fisher Scientific) was used to replace the potassium hydroxide gradient with pure water before entering the mass spectrometer. The mass spectrometer was operated in electrospray ionization (ESI) negative mode. A full mass scan was performed with the following settings: range, 70 to 900 *m/z*; resolution, 70,000; automatic gain control target, 3E6; and maximum ion injection time, 100 ms. The ion source parameters were as follows: spray voltage, 3 kV; transfer temperature, 320°C; S-lens level, 50; heater temperature, 300°C; sheath gas, 36; and aux gas, 10. The levels of cationic metabolites were quantified by LC-MS/MS. The samples were separated on a Discovery HS F5-3 column (2.1 mm i.d. by 150 mm long, 3-μm particle size, Sigma-Aldrich) at a flow rate of 0.25 ml/min with a step gradient of mobile phase A (0.1% formic acid) and mobile phase B (0.1% acetonitrile) according to the following protocol: 100:0 (0 to 5 min), 75:25 (5 to 11 min), 65:35 (11 to 15 min), 5:95 (15 to 20 min), and 100:0 (20 to 25 min). The column temperature was set at 40°C. An LCMS-8060 triple quadrupole mass spectrometer (Shimadzu) equipped with an ESI ion source was operated in positive and negative ESI, multiple-reaction monitoring mode. The relative levels of each metabolite were normalized to protein levels of duplicate samples as measured by BCA assay. For glucose or glutamine labeling, DMEM without glucose and glutamine (Sigma-Aldrich, D5030) was supplemented with [U-^13^C]glucose (Sigma-Aldrich, 389374) and glutamine (Sigma-Aldrich, G-9723), or glucose (Wako, 041-00595) and [^13^C_5_,^15^N_2_]glutamine (Taiyo Nippon Sanso, B06-0008). For palmitate labeling, [U-^13^C]palmitic acid (MedChemExpress, HY-N0830S6) was dissolved in 0.1 M NaOH at 70°C and added to 0.9% NaCl solution containing fatty acid–free BSA (Wako, 017-15146) at 45°C to obtain 5 mM palmitate solution (palmitate: BSA = 6: 1). The cells were incubated in serum-free DMEM supplemented with 100 μM palmitate for 16 hours. Unlabeled palmitic acid (Tokyo Chemical Industry, P0002) was used as a control.

### Oxygen consumption analysis

The cells were seeded in XF24 (Agilent Technologies, 100867-100) or XF96 (Agilent Technologies, 102601-100) plates. Before measurements, the medium was replaced with Seahorse XF DMEM medium (Agilent Technologies, 103575-100) supplemented with 5 mM glucose and 2 mM glutamine. Oxygen consumption rates were measured using an XF24 or XF96 extracellular flux analyzer (Agilent Technologies) and the following inhibitors: 3 μM oligomycin A (Sigma-Aldrich, 75351); 1 μM carbonyl cyanide 4-(trifluoromethoxy)phenylhydrazone (Cayman Chemical, 15218); 2 μM rotenone (Sigma-Aldrich, R8875); and 4 μM antimycin A (Sigma-Aldrich, A8674). Oxygen consumption rates were normalized to protein levels as measured by BCA assay.

### Fluorescence measurement of FAO activity

The cells were seeded in black glass-bottom 96-well plates (Matsunami Glass, GP96000), washed twice with Hanks’ balanced solution [HBS: 20 mM Hepes, 107 mM NaCl, 6 mM KCl, 1.2 mM MgSO_4_, 2 mM CaCl_2_, and 11.5 mM glucose (pH 7.4)] and incubated with 20 μM FAOBlue (Funakoshi, FDV-0033) in HBS for 2 hours at 37°C. To evaluate and subtract autofluorescence, the cells were also incubated in HBS without FAOBlue. Fluorescence was measured with a Varioskan microplate reader (Ex/Em = 405/460 nm, Thermo Fisher Scientific). To normalize measurements to cell number, the cells were fixed with methanol for 5 min, stained with Hoechst 33258 (1:2000) in PBS for 30 min, and counted with a CellInsight NXT automated microscope.

### Lipid droplet staining

The cells were seeded on glass coverslips, fixed with 4% paraformaldehyde in PBS for 10 min, and incubated with 1 μM BODIPY 493/503 (Cayman Chemical, 25892) and 4′,6-diamidino-2-phenylindole (Dojindo, D523, 1:2000). Images were acquired using a TCS SP5 confocal microscope with a 63× oil immersion objective. Lipid droplet size was quantified using the “analyze particles” tool of ImageJ software.

### ChIP-qPCR

ChIP was performed using anti-H3K27ac (Active Motif, 39133), anti-H3K9ac (Sigma-Aldrich, 07-352), and anti-histone H3 (Abcam, ab1791) antibodies and an enzymatic ChIP kit (Cell Signaling Technology, 9003) according to the manufacturer’s protocol. qPCR was performed with the following primers: primer pair 1, forward, 5′-GGATTCTAAGCCAACATCATTTC-3′, reverse, 5′-TGGATAGTTTTGACAATTTTTAATGG-3′; primer pair 2, forward, 5′-TCCCCTAACTCCCTCACAAA-3′, reverse, 5′-TCCTCATTTTTGAACAGAGGTG-3′; primer pair 3, forward, 5′-AGGATTCCTTTTGGAGAGTCG-3′, reverse, 5′-TACACGCAGGAGGGGAAG-3′; and primer pair 4, forward, 5′-TCCCCTCTGATATTATTAAAGATTGC-3′, reverse, 5′-GTTGAATCACATATCAGGTGAAGAAT-3′. The percent input was calculated using the ΔΔ*C*_t_ method. ChIP-seq data (GSE74328) (*69*) were downloaded from the Gene Expression Omnibus database and visualized with Integrative Genomics Viewer (Broad Institute).

### Statistics

R software and Microsoft Excel were used for statistical analyses. Statistical tests and sample size are reported in the figure legends. *P* < 0.05 was considered statistically significant. No statistical method was used to predetermine the sample size.

## Supplementary Materials

### The PDF file includes:

Figs. S1 to S12 Uncropped gels Legends for tables S1 and S2 Legends for movies S1 to S4
