# Supplementary material for: Mitochondrial fatty acid oxidation drives senescence
Source: Sci Adv. 2024 Oct 25;10(43):eado5887. doi: 10.1126/sciadv.ado5887 (PMC11506141; doi:10.1126/sciadv.ado5887)

Science Advances
AAAS

Supplementary Materials for

Mitochondrial fatty acid oxidation drives senescence

Shota Yamauchi *et al.*

Corresponding author: Shota Yamauchi, yamauchi-s@umin.ac.jp; Hidenori Ichijo, ichijo@g.ecc.u-tokyo.ac.jp

Sci. Adv. **10**, eado5887 (2024)
DOI: 10.1126/sciadv.ado5887

The PDF file includes:

Figs. S1 to S12
Uncropped gels
Legends for tables S1 and S2
Legends for movies S1 to S4

Other Supplementary Material for this manuscript includes the following:

Tables S1 and S2
Movies S1 to S4

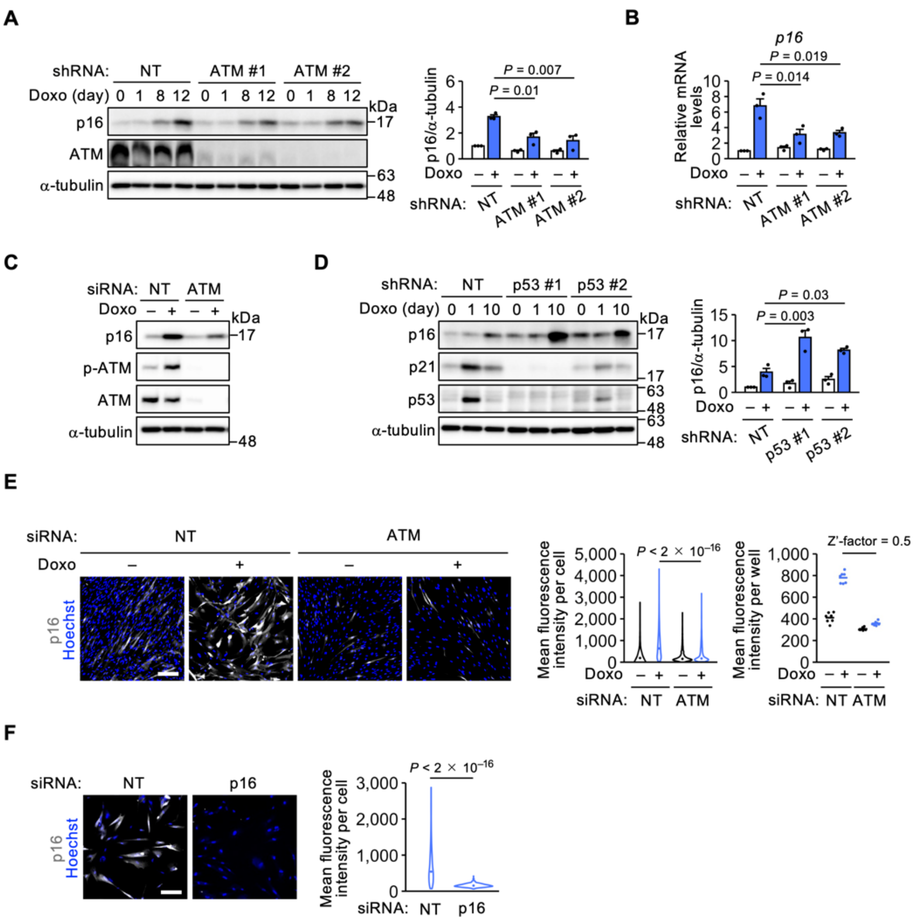

Fig. S1. p16 expression requires ATM but not p53. (**A**) Immunoblot analysis of IMR-90 cells infected with lentiviruses encoding nontargeting (NT) and ATM shRNAs and treated with doxorubicin (Doxo). *n* = 3 independent experiments. (**B**) qPCR analysis of IMR-90 cells infected with lentiviruses encoding NT and ATM shRNAs and treated with Doxo for 12 days. *n* = 3 independent experiments. (**C**) Immunoblot analysis of IMR-90 cells transfected with NT and ATM siRNAs and treated with Doxo for 12 days. (**D**) Immunoblot analysis of IMR-90 cells infected with lentiviruses encoding NT and p53 shRNAs and treated with Doxo. *n* = 3 independent experiments. (**E**) Left, immunofluorescence analysis of IMR-90 cells transfected with NT and ATM siRNAs (siGENOME) and treated with Doxo for 10 days. p16 is shown in gray. Scale bar, 200 µm. Center, distribution of the mean fluorescence intensity per cell. Dot, median. *n* = 3,405 (siNT), 1,099 (siNT + Doxo), 2,926 (siATM), 1,306 (siATM + Doxo) cells. Right, mean fluorescence intensity per well. Line, mean. *n* = 7 wells. (**F**) Left, immunofluorescence analysis of Doxo-induced senescent cells transfected with NT and p16 siRNAs (siGENOME). p16 is shown in gray. Scale bar, 200 µm. Right, distribution of the mean fluorescence intensity per cell. *n* = 704 (siNT), 755 (sip16) cells. Data are mean ± s.e.m. Statistical analysis was performed using Dunnett's multiple comparison test (A, B, D) and the Wilcoxon rank-sum test with Bonferroni correction (E, center, F).

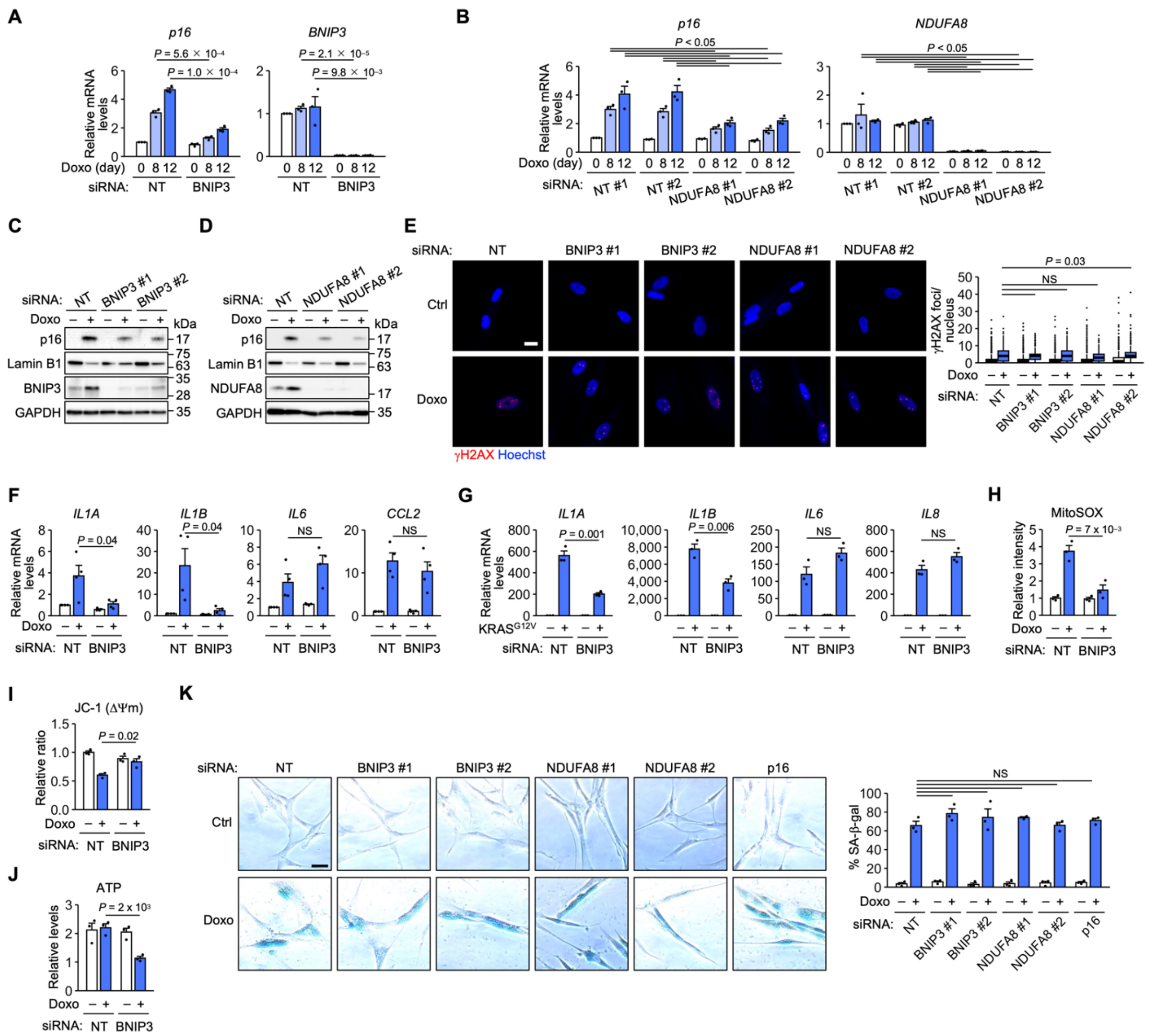

Fig. S2. BNIP3 is involved in several features of senescence. (**A** and **B**) qPCR analysis of IMR-90 cells transfected with the indicated siRNAs and treated with doxorubicin (Doxo) for 12 days. n = 3 independent experiments. (**C** and **D**) Immunoblot analysis of HUVECs transfected with the indicated siRNAs and treated with 50 ng/ml Doxo. Medium was changed every 3 days. (**E**) Left, immunofluorescence analysis of IMR-90 cells transfected with the indicated siRNAs and treated with Doxo for 12 days. γH2AX is shown in red. Scale bar, 20 μm. Right, distribution of the number of γH2AX foci. Center line, median; box limits, upper and lower quartiles; whiskers, 1.5× interquartile range; points, outliers. n = 4,494 (siNT), 501 (siNT + Doxo), 2,681 (siBNIP3 #1), 1,269 (siBNIP3 #1 + Doxo), 4,233 (siBNIP3 #2), 435 (siBNIP3 #2 + Doxo), 2,489 (siNDUFA8 #1), 644 (siNDUFA8 #1 + Doxo), 2,926 (siNDUFA8 #2), 981 (siNDUFA8 #2 + Doxo) cells. (**F**) qPCR analysis of IMR-90 cells transfected with BNIP3 siRNA #1 and treated with Doxo for 12 days. n = 4 independent experiments. (**G**) qPCR analysis of IMR-90 cells expressing ER-KRAS^{G12V} for 10 days. n = 3 independent experiments. (**H**) Mitochondrial ROS levels in IMR-90 cells transfected with the indicated siRNAs and treated with Doxo for 12 days. n = 3 biological replicates. (**I**) Mitochondrial membrane potential. n = 3 biological replicates. (**J**) ATP levels measured by mass spectrometry. n = 3 biological replicates. (**K**) SA-β-gal staining in IMR-90 cells

transfected with the indicated siRNAs and treated with Doxo. Scale bar, 50 µm. $n = 3$ biological replicates. Data are mean ± s.e.m. Statistical analysis was performed using unpaired two-tailed Student's *t*-test (A, F, G, H, I, J), Tukey's multiple comparison test (B), the Wilcoxon rank-sum test with Bonferroni correction (E), and Dunnett's multiple comparison test (K). NS, not significant.

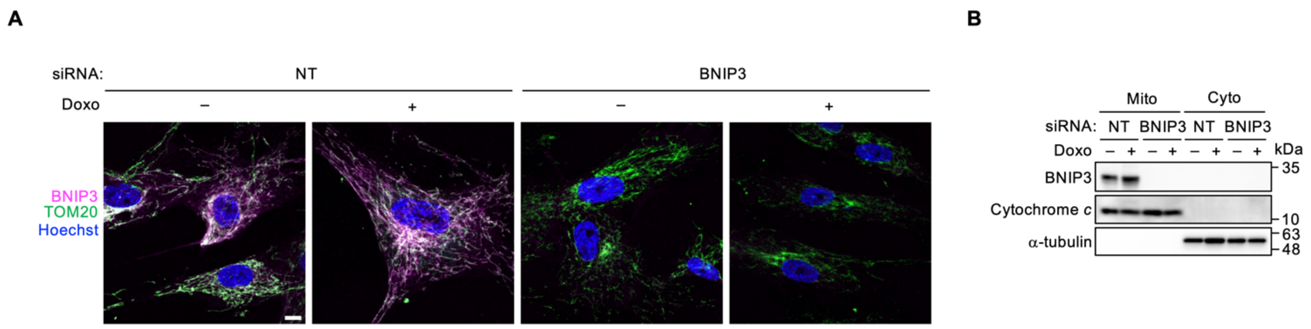

Fig. S3. BNIP3 is localized to mitochondria. (**A**) Immunofluorescence analysis of IMR-90 cells transfected with nontargeting (NT) and BNIP3 #1 siRNAs and treated with doxorubicin (Doxo) for 12 days. Magenta, BNIP3; green, TOM20. Scale bar, 10 μm. (**B**) Subcellular fractionation of IMR-90 cells treated with Doxo for 12 days. The same amount of protein was loaded in each lane.

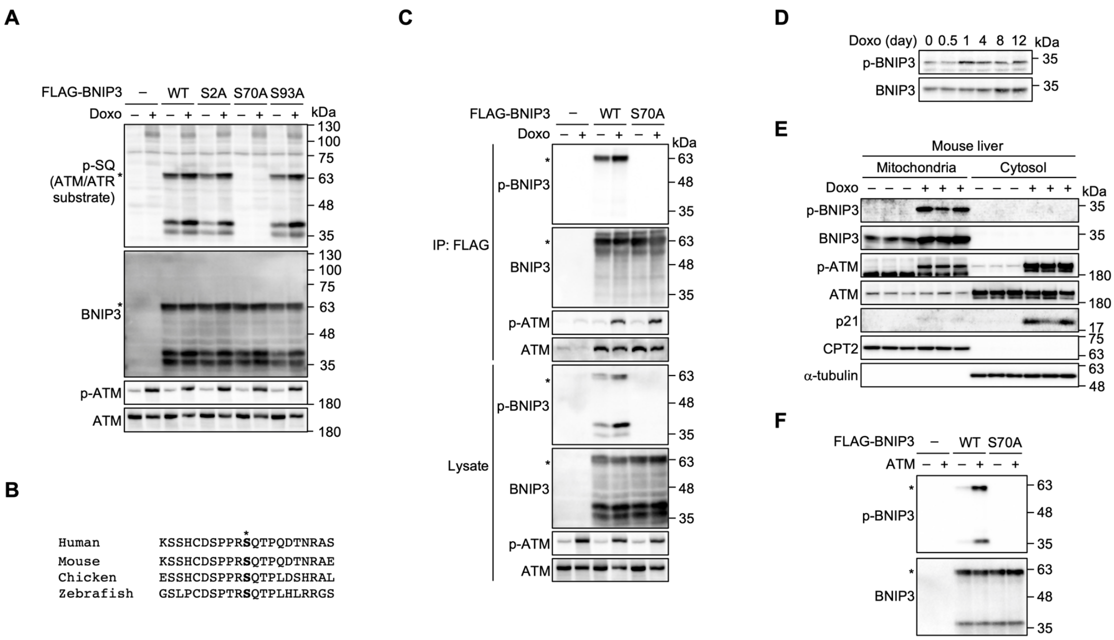

Fig. S4. BNIP3 is phosphorylated at S70. (**A**) Immunoblot analysis of HEK293T cells transfected with the indicated plasmids and treated with doxorubicin (Doxo) for 24 h. The asterisks denote the BNIP3 dimer. (**B**) Alignment of BNIP3 amino acid sequences from the indicated species. The asterisk denotes S70. (**C**) Immunoprecipitation (IP) of FLAG-BNIP3 from HEK293T cells treated with Doxo for 24 h. The asterisks denote the BNIP3 dimer. (**D**) Immunoblot analysis of IMR-90 cells. (**E**) Immunoblot analysis of mitochondrial and cytosolic fractions of mouse liver 24 hours after intraperitoneal injection of 10 mg/kg Doxo. *n* = 3 (Ctrl), 3 (Doxo) mice. (**F**) In vitro kinase assay using recombinant ATM.

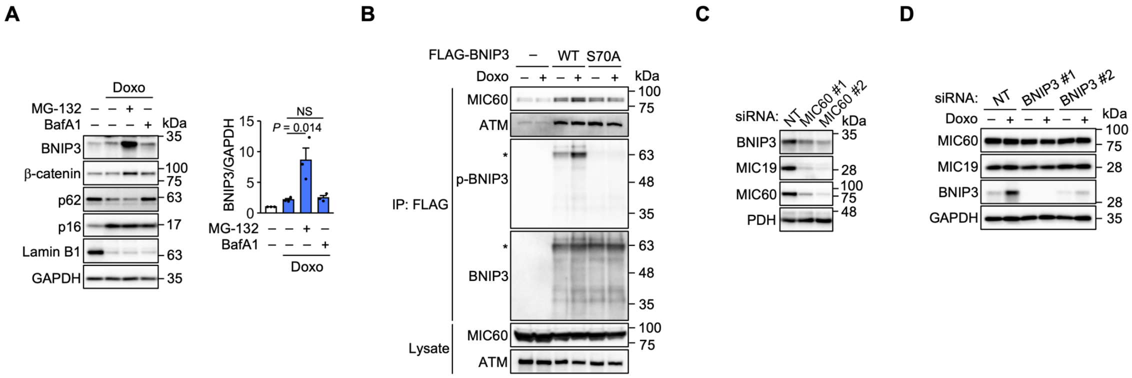

Fig. S5. BNIP3 protein stability depends on MIC60. (**A**) Immunoblot analysis of IMR-90 cells treated with doxorubicin (Doxo) for 12 days and then with MG-132 and bafilomycin A1 (BafA1) for 10 h. $n = 3$ independent experiments. Data are mean ± s.e.m. Statistical analysis was performed using Dunnett's multiple comparison test. NS, not significant. (**B**) Immunoprecipitation (IP) of FLAG-BNIP3 from HEK293T cells treated with Doxo for 24 h. The asterisks denote the BNIP3 dimer. (**C** and **D**) Immunoblot analysis of IMR-90 cells transfected with the indicated siRNAs and treated with Doxo for 12 days.

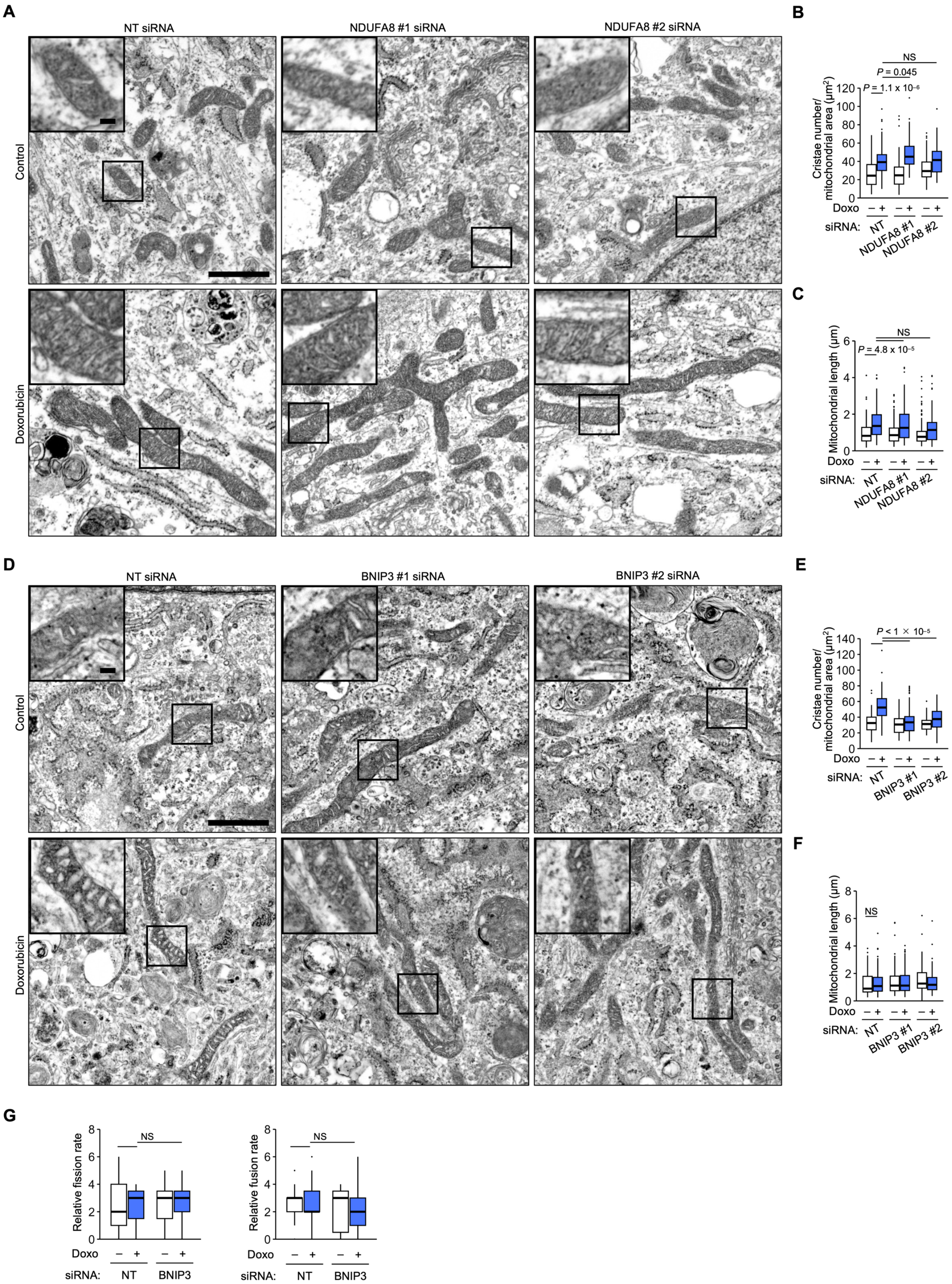

Fig. S6. NDUFA8 is dispensable for mitochondrial structural changes associated with senescence. (**A**) Electron microscopy of IMR-90 cells transfected with nontargeting (NT) and NDUFA8 siRNAs and treated with Doxo for 12 days. Scale bar, 1 μm; inset scale bar, 100 nm. (**B**

and **C**) Quantification of the number of cristae per mitochondrial area (B) and mitochondrial length (C). *n* = 145 (siNT), 84 (siNT + Doxo), 96 (siNDUFA8 #1), 64 (siNDUFA8 #1 + Doxo), 85 (siNDUFA8 #2), 84 (siNDUFA8 #2 + Doxo) mitochondria. Center line, median; box limits, upper and lower quartiles; whiskers, 1.5× interquartile range; points, outliers. (**D**) Electron microscopy of HUVECs transfected with NT and BNIP3 siRNAs and treated with Doxo for 12 days. Scale bar, 1 μm; inset scale bar, 100 nm. (**E** and **F**) Quantification of the number of cristae per mitochondrial area (E) and mitochondrial length (F). *n* = 78 (siNT), 92 (siNT + Doxo), 63 (siBNIP3 #1), 115 (siBNIP3 #1 + Doxo), 48 (siBNIP3 #2), 62 (siBNIP3 #2 + Doxo) mitochondria. (**G**) Mitochondrial fission and fusion rates in IMR-90 cells transfected with NT and BNIP3 #1 siRNAs and treated with Doxo for 12 days. The number of mitochondrial fissions and fusions per 10 min in a cellular area (16.35 μm x 16.35 μm) without dense mitochondria was counted. See also Movies S1 to S4. Statistical analysis was performed using the Wilcoxon rank-sum test with Bonferroni correction. NS, not significant.

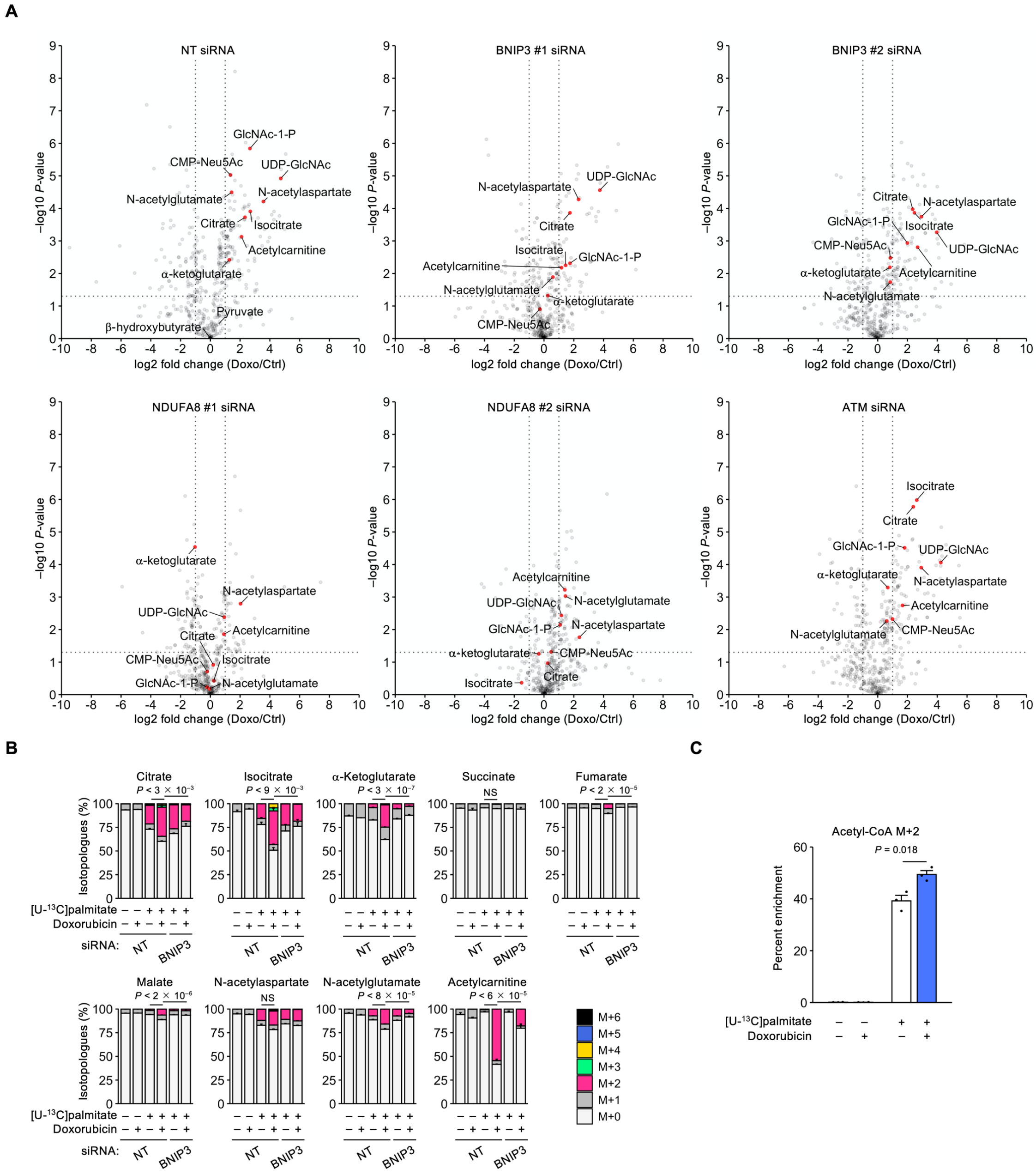

Fig. S7. Levels of acetyl-CoA-derived metabolites increase during senescence. (**A**) Volcano plot of metabolite level changes upon doxorubicin (Doxo) treatment for 12 days in IMR-90 cells transfected with the indicated siRNAs. Metabolite levels were normalized to protein levels. $n = 3$ biological replicates. (**B**) Distributions of the isotopologues containing ^{13}C derived from labeled palmitate in control and doxorubicin-induced senescent HUVECs transfected with the indicated siRNAs. (**C**) Percentage of acetyl-CoA M+2 in control and Doxo-induced senescent IMR-90 cells after 2 h incubation with labeled palmitate. Statistical analysis was performed using Dunnett's multiple comparison test (B) and unpaired two-tailed Student's *t*-test (C). NS, not significant.

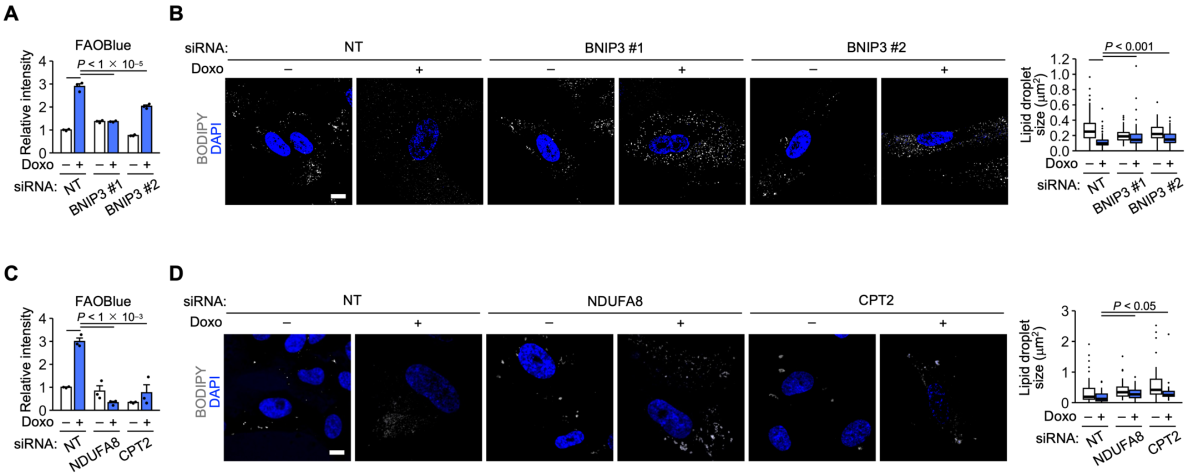

Fig. S8. FAO activity increases during senescence. (**A**) FAO activity assay using IMR-90 cells transfected with nontargeting (NT) and BNIP3 siRNAs and treated with doxorubicin (Doxo) for 12 days. n = 3 biological replicates. (**B**) Left, lipid droplet staining in IMR-90 cells treated with Doxo for 14 days. Lipid droplets are shown in gray. Scale bar, 10 µm. Right, quantification of lipid droplet size. n = 171 (siNT), 85 (siNT + Doxo), 53 (siBNIP3 #1), 121 (siBNIP3 #1 + Doxo), 102 (siBNIP3 #2), 79 (siBNIP3 #2 + Doxo) cells. Center line, median; box limits, upper and lower quartiles; whiskers, 1.5× interquartile range; points, outliers. (**C**) FAO activity assay using IMR-90 cells transfected with the indicated siRNAs and treated with Doxo for 12 days. n = 3 biological replicates. (**D**) Left, lipid droplet staining in IMR-90 cells treated with Doxo for 14 days. Lipid droplets are shown in gray. Scale bar, 10 µm. Right, quantification of lipid droplet size. n = 49 (siNT), 38 (siNT + Doxo), 47 (siNDUFA8 #1), 31 (siNDUFA8 #1 + Doxo), 39 (siCPT2 #1), 31 (siCPT2 #1 + Doxo) cells. Data are mean ± s.e.m. Statistical analysis was performed using Dunnett's multiple comparison test (A, C) and the Wilcoxon rank-sum test with Bonferroni correction (B, D).

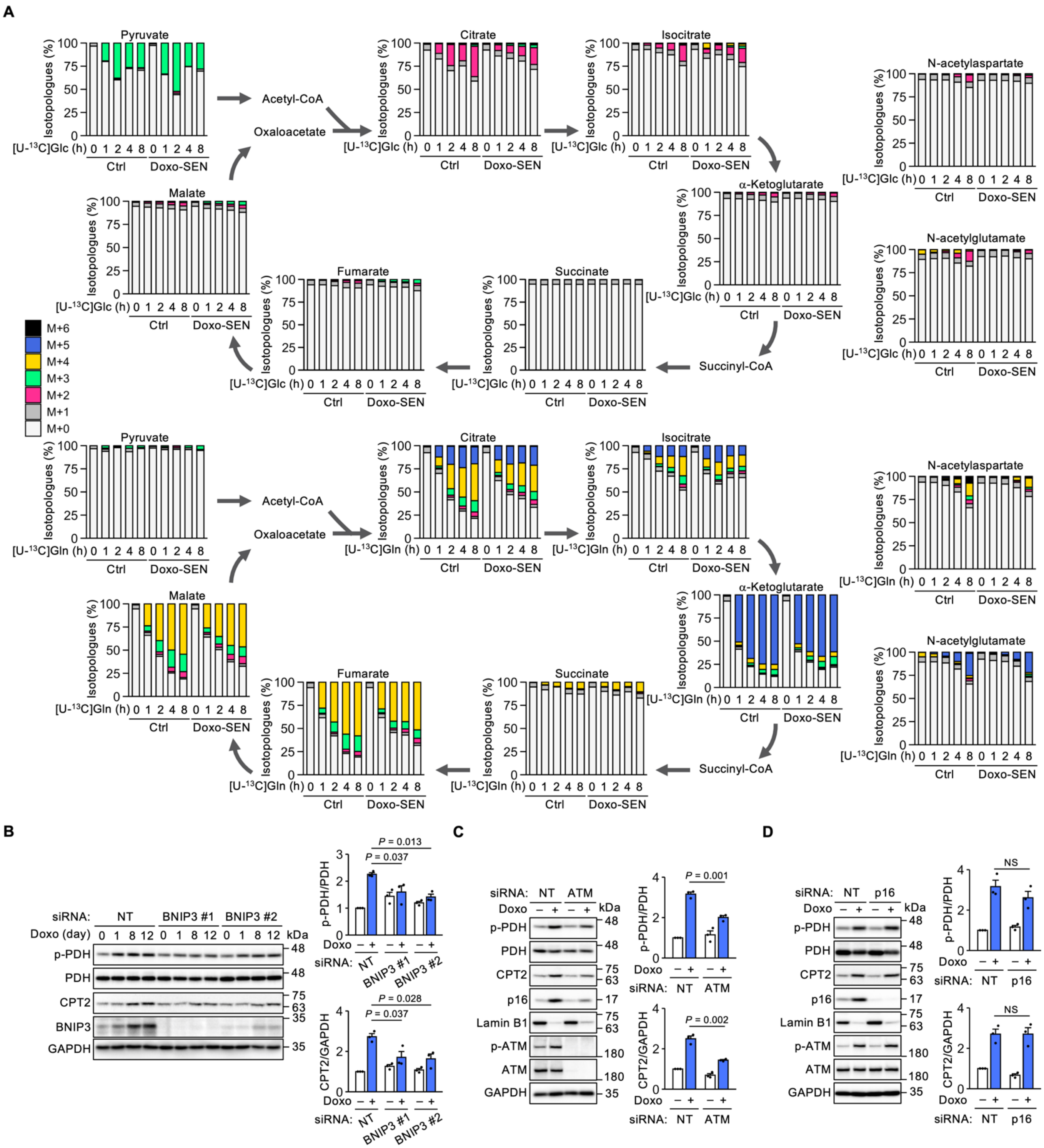

Fig. S9. Glucose utilization decreases during senescence. (**A**) Distributions of the isotopologues containing ^{13}C derived from labeled glucose (Glc) (top) or glutamine (Gln) (bottom) in control and doxorubicin-induced senescent (Doxo-SEN) cells. (**B** to **D**) Immunoblot analysis of IMR-90 cells transfected with the indicated siRNAs and treated with Doxo for 12 days. *n* = 3 independent experiments. Data are mean ± s.e.m. Statistical analysis was performed using Dunnett's multiple comparison test (B) and unpaired two-tailed Student's *t*-test (C, D). NS, not significant.

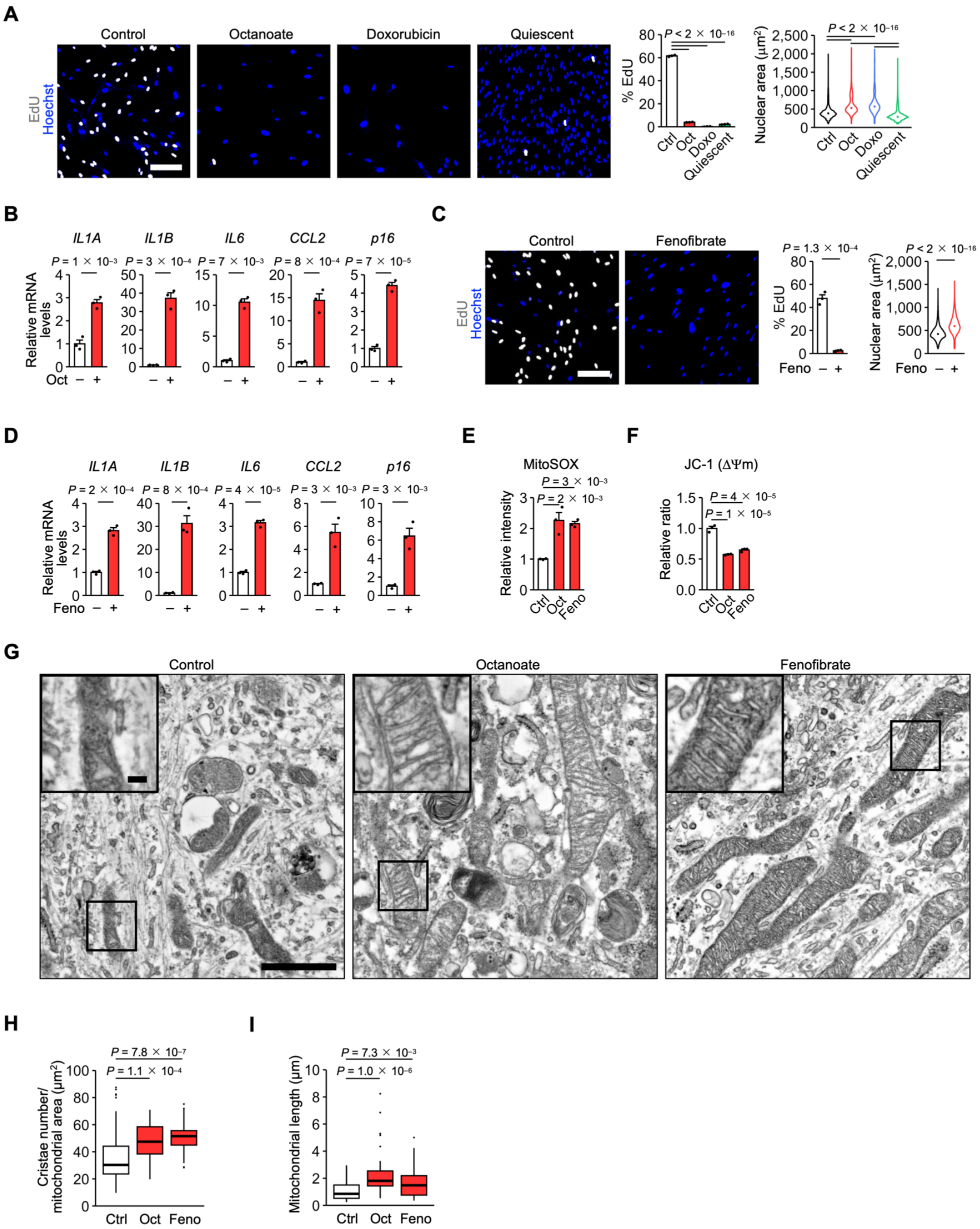

Fig. S10. Pharmacological activation of FAO increases the expression of inflammatory cytokines and chemokines. (**A**) Left, EdU assay using IMR-90 cells treated with Octanoate (Oct) or doxorubicin (Doxo) for 12 days or made quiescent by confluence. EdU is shown in gray. Scale bar, 200 µm. Center, percentage of EdU-positive cells. n = 3 biological replicates. Right, distribution of the nuclear area. Dot, median. n = 7,790 (Ctrl), 3,748 (Oct), 967 (Doxo), 17,185 (quiescent) cells. (**B**) qPCR analysis of IMR-90 cells treated with Oct for 12 days. n = 3 biological replicates. (**C**) Left, EdU assay using IMR-90 cells treated with fenofibrate (Feno) for 12 days. EdU is shown in gray. Scale bar, 200 µm. Center, percentage of EdU-positive cells. n = 3 biological replicates. Right, distribution of the nuclear area. n = 7,287 (Ctrl), 3,652 (Feno) cells. (**D**) qPCR analysis of IMR-90 cells treated with Feno for 12 days. n = 3 biological replicates. (**E**) Mitochondrial ROS levels in IMR-90 cells treated with Oct or Feno for 12 days. n = 3 biological replicates. (**F**) Mitochondrial membrane potential. n = 3 biological replicates. (**G**) Electron microscopy of IMR-90 cells treated with Oct or Feno for 12 days. Scale bar, 1 µm; inset scale bar,

100 nm. (**H** and **I**) Quantification of the number of cristae per mitochondrial area (H) and mitochondrial length (I). *n* = 61 (Ctrl), 46 (Oct), 55 (Feno) mitochondria. Center line, median; box limits, upper and lower quartiles; whiskers, 1.5× interquartile range; points, outliers. Data are mean ± s.e.m. Statistical analysis was performed using Dunnett's multiple comparison test (A, center, E, F), the Wilcoxon rank-sum test with Bonferroni correction (A, right, C, right, H, I), and unpaired two-tailed Student's *t*-test (B, C, center, D).

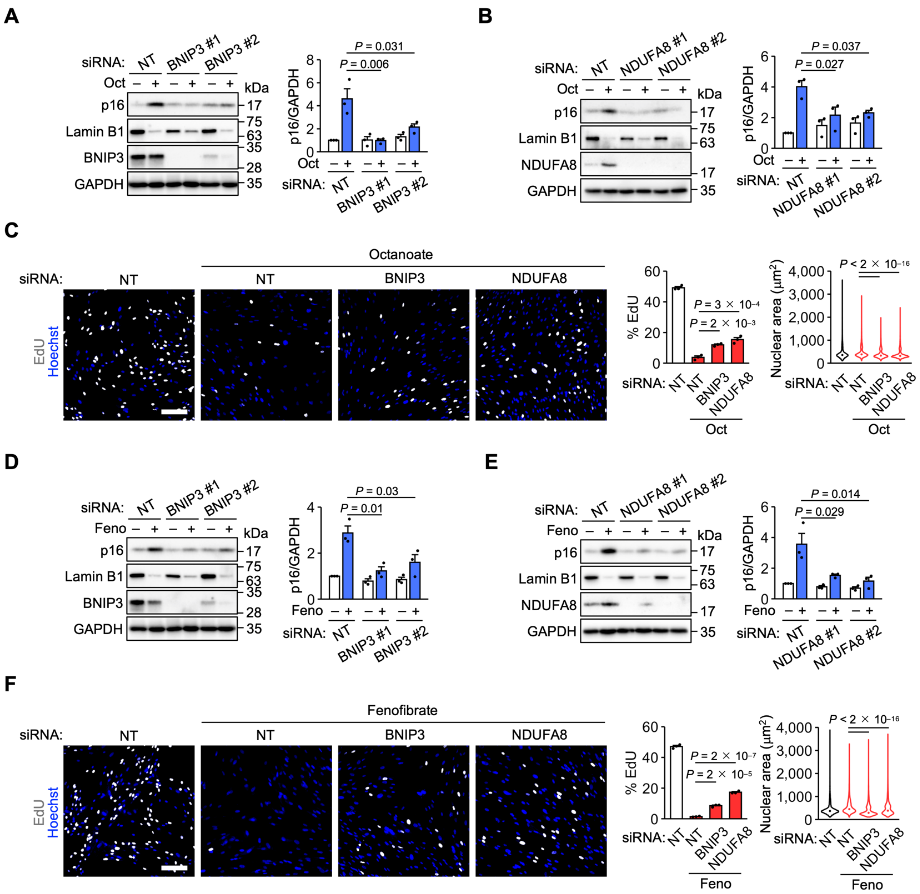

Fig. S11. BNIP3 is involved in FAO-induced senescence. (**A** and **B**) Immunoblot analysis of IMR-90 cells transfected with the indicated siRNAs and treated with octanoate (Oct). *n* = 3 independent experiments. (**C**) Left, EdU assay using IMR-90 cells transfected with the indicated siRNAs and treated with Oct for 12 days. EdU is shown in gray. Scale bar, 200 µm. Center, percentage of EdU-positive cells. *n* = 3 biological replicates. Right, distribution of the nuclear area. Dot, median. *n* = 9,736 (siNT), 12,584 (siNT + Doxo), 14,024 (siBNIP3 + Doxo), 16,760 (siNDUFA8 + Doxo) cells. (**D** and **E**) Immunoblot analysis of IMR-90 cells transfected with the indicated siRNAs and treated with fenofibrate (Feno). *n* = 3 independent experiments. (**F**) Left, EdU assay using IMR-90 cells transfected with the indicated siRNAs and treated with Feno for 12 days. EdU is shown in gray. Scale bar, 200 µm. Center, percentage of EdU-positive cells. *n* = 3 biological replicates. Right, distribution of the nuclear area. *n* = 9,464 (siNT), 9,966 (siNT + Doxo), 16,306 (siBNIP3 + Doxo), 12,642 (siNDUFA8 + Doxo) cells. Data are mean ± s.e.m. Statistical analysis was performed using Dunnett's multiple comparison test (A, B, C, center, D, E, F, center) and the Wilcoxon rank-sum test with Bonferroni correction (C, right, F, right).

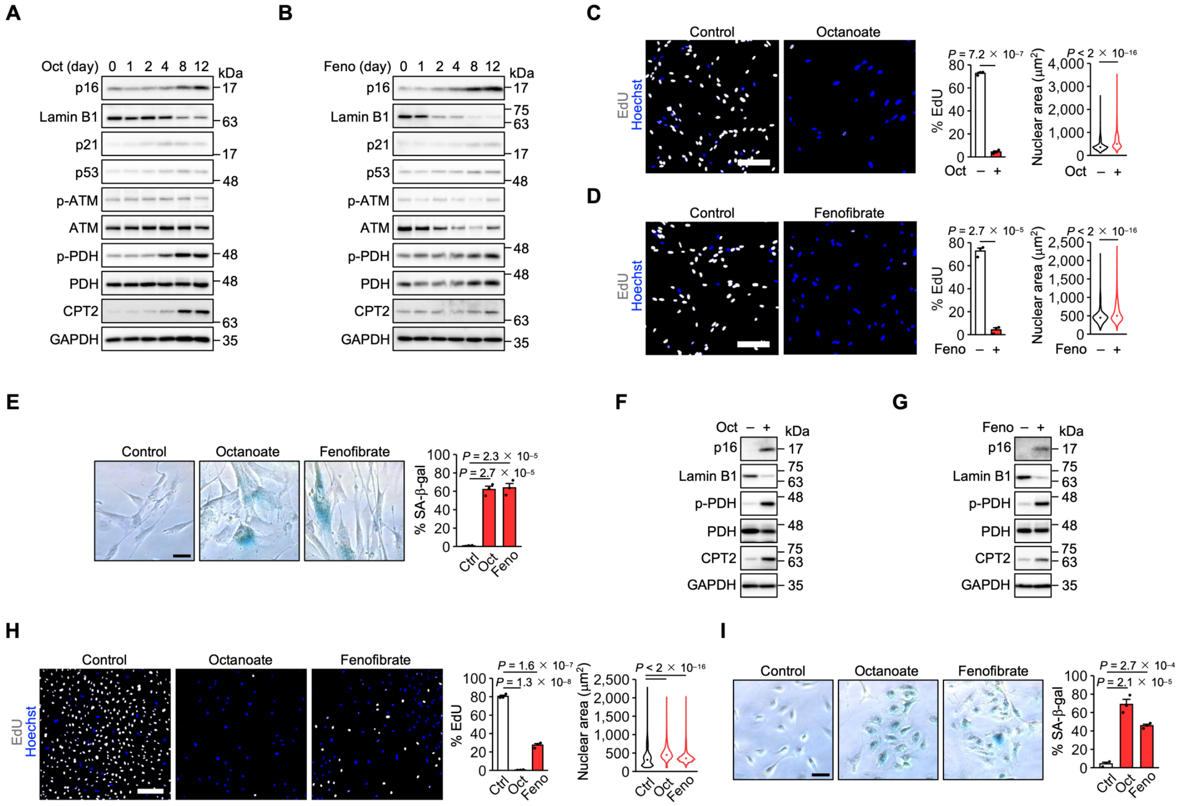

Fig. S12. Pharmacological activation of FAO induces senescence in TIG-3 cells and HUVECs. (**A** and **B**) Immunoblot analysis of TIG-3 cells treated with octanoate (Oct) (A) or fenofibrate (B). (**C**) Left, EdU assay using TIG-3 cells treated with Oct for 12 days. EdU is shown in gray. Scale bar, 200 µm. Center, percentage of EdU-positive cells. n = 3 biological replicates. Right, distribution of the nuclear area. Dot, median. n = 9,081 (Ctrl), 4,509 (Oct) cells. (**D**) Left, EdU assay using TIG-3 cells treated with Feno for 12 days. EdU is shown in gray. Scale bar, 200 µm. Center, percentage of EdU-positive cells. n = 3 biological replicates. Right, distribution of the nuclear area. n = 3,451 (Ctrl), 6,947 (Feno). (**E**) SA-β-gal staining in TIG-3 cells treated with Oct or Feno for 12 days. Scale bar, 50 µm. n = 3 biological replicates. (**F** and **G**) Immunoblot analysis of HUVECs treated with 2.5 mM Oct (F) or 25 µM Feno (G). (**H**) Left, EdU assay using HUVECs treated with 1 mM Oct and 25 µM Feno for 12 days. EdU is shown in gray. Scale bar, 200 µm. Center, percentage of EdU-positive cells. n = 3 biological replicates. Right, distribution of the nuclear area. n = 14,830 (Ctrl), 3,184 (Oct), 5,043 (Feno) cells. (**I**) SA-β-gal staining in HUVECs treated with 2.5 mM Oct or 50 µM Feno for 12 days. Scale bar, 50 µm. n = 3 biological replicates. Data are mean ± s.e.m. Statistical analysis was performed using unpaired two-tailed Student's t-test (C, center, D, center), the Wilcoxon rank-sum test with Bonferroni correction (C, right, D, right, H, right), and Dunnett's multiple comparison test (E, H, center, I).

Table S1. Robust Z-scores of the screened siRNAs.
Table S2. BNIP3-interacting proteins identified by mass spectrometry.
Movie S1. Mitochondrial dynamics in IMR-90 cells transfected with NT siRNA. Images were acquired every 15s. Video rate, 4 f/s. Scale bar, 5 μm. Magenta, fission; green, fusion.
Movie S2. Mitochondrial dynamics in IMR-90 cells transfected with NT siRNA and treated with doxorubicin for 12 days. Images were acquired every 15s. Video rate, 4 f/s. Scale bar, 5 μm. Magenta, fission; green, fusion.
Movie S3. Mitochondrial dynamics in IMR-90 cells transfected with BNIP3 siRNA. Images were acquired every 15s. Video rate, 4 f/s. Scale bar, 5 μm. Magenta, fission; green, fusion.
Movie S4. Mitochondrial dynamics in IMR-90 cells transfected with BNIP3 siRNA and treated with doxorubicin for 12 days. Images were acquired every 15s. Video rate, 4 f/s. Scale bar, 5 μm. Magenta, fission; green, fusion.

Uncropped gels

Fig. 1C

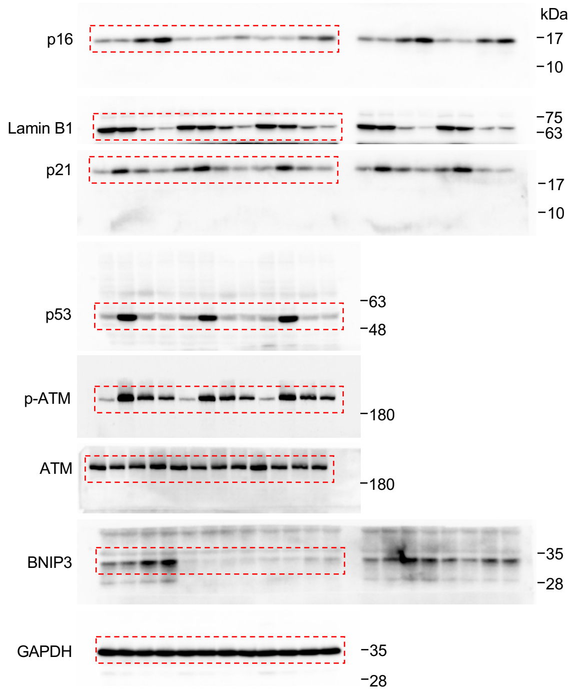

Fig. 1D

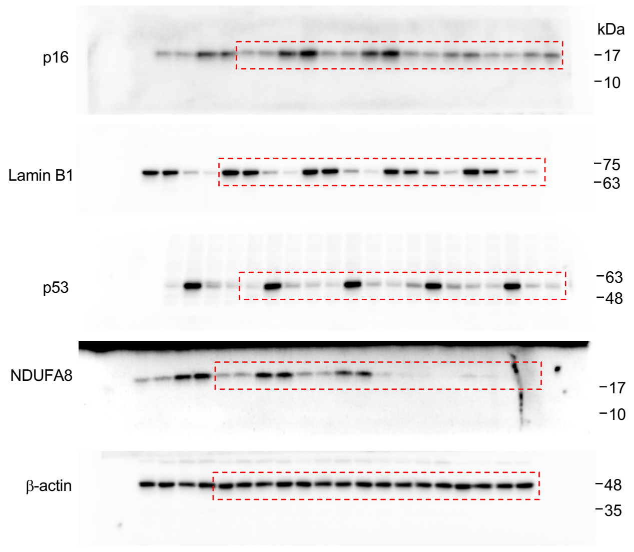

Fig. 1E

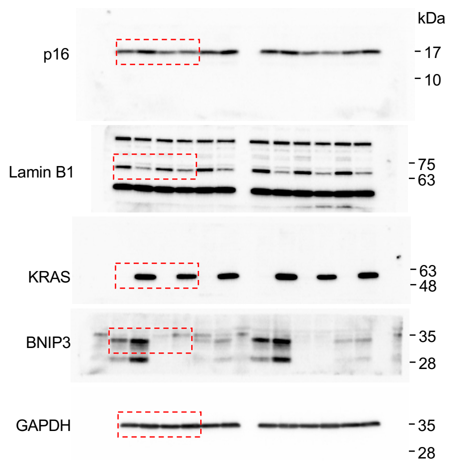

Fig. 2B

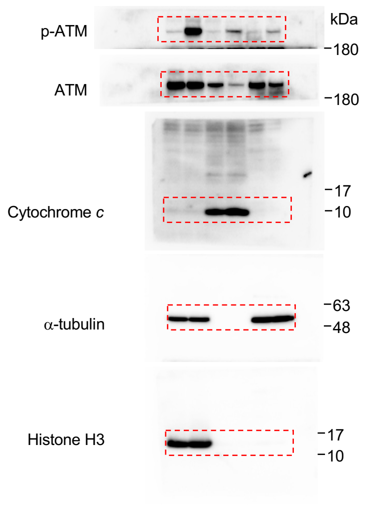

Fig. 2E

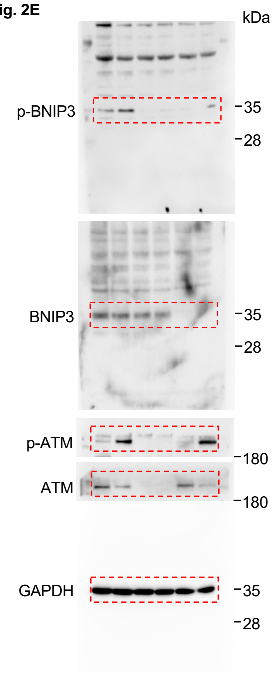

Fig. 2F

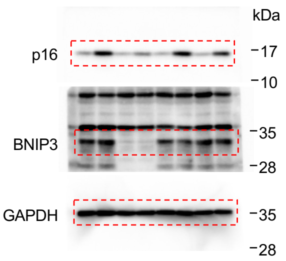

Fig. 4G

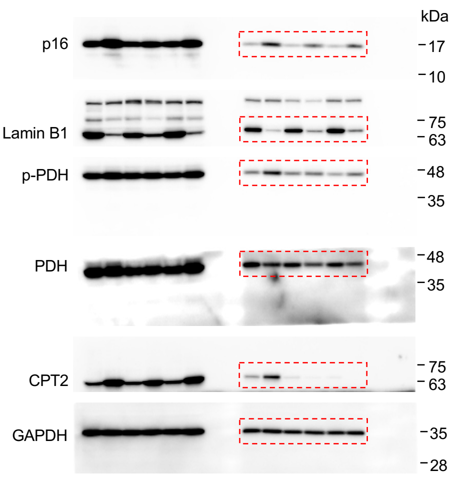

Fig. 4H

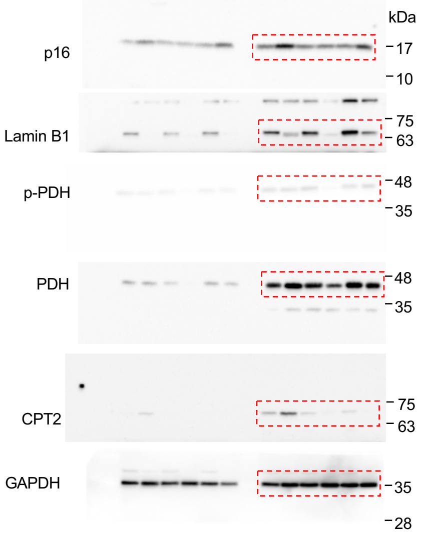

Fig. 5A

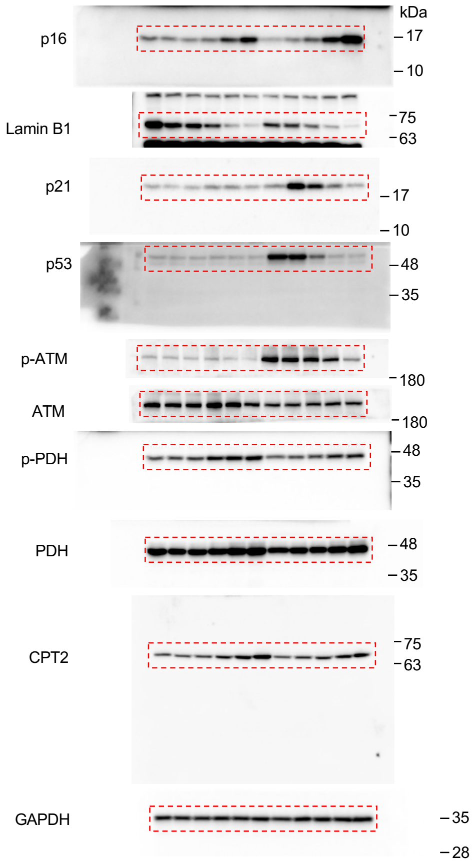

Fig. 6A

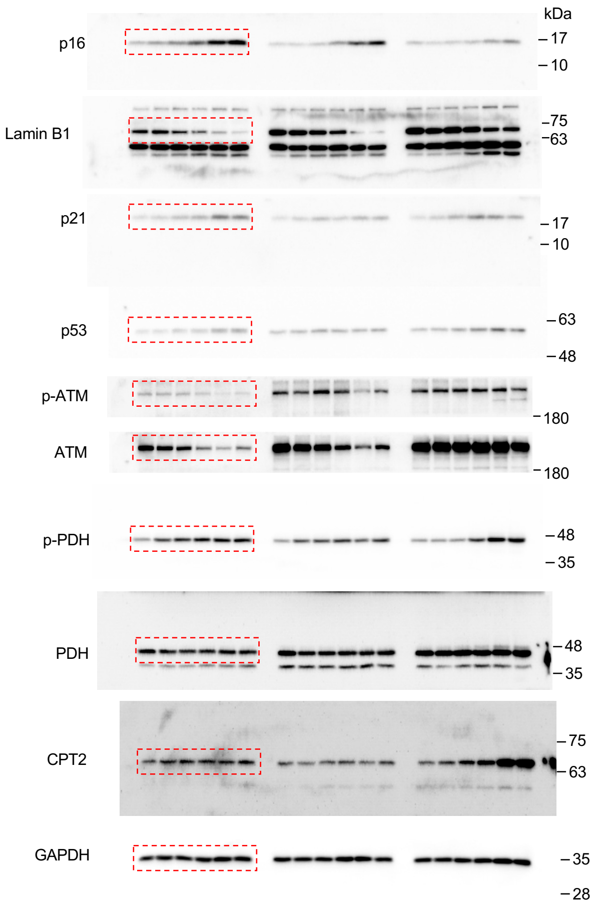

Fig. 6B

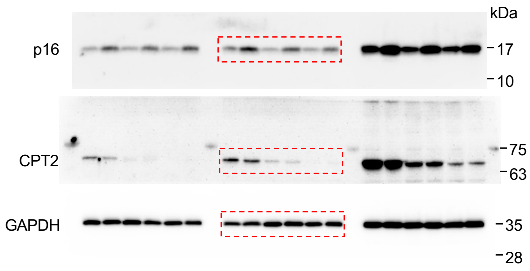

Fig. 7I

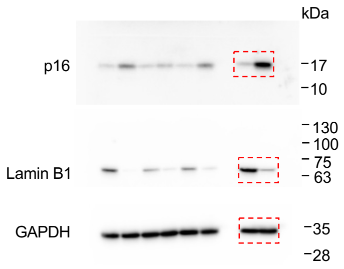

Fig. 7J

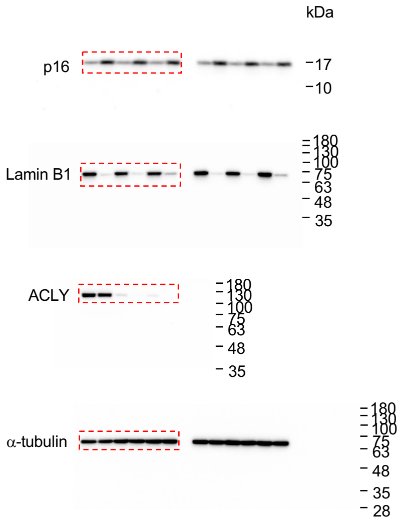

Fig. 7K

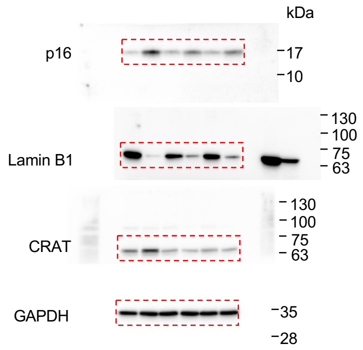

Fig. S1A

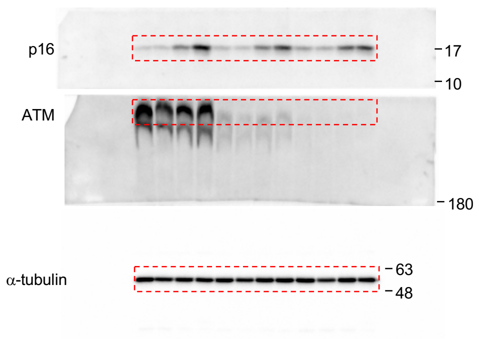

Fig. S1C

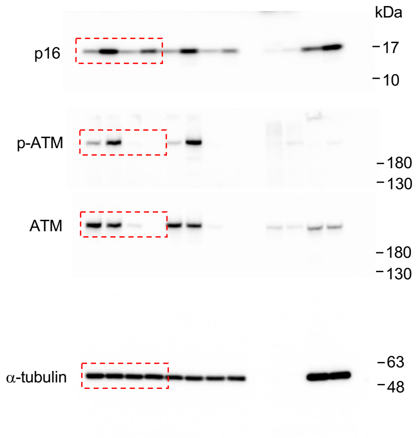

Fig. S1D

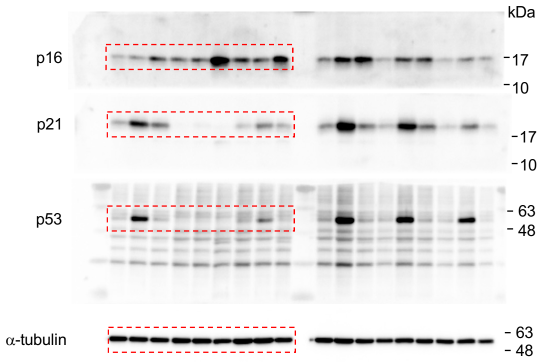

Fig. S2C

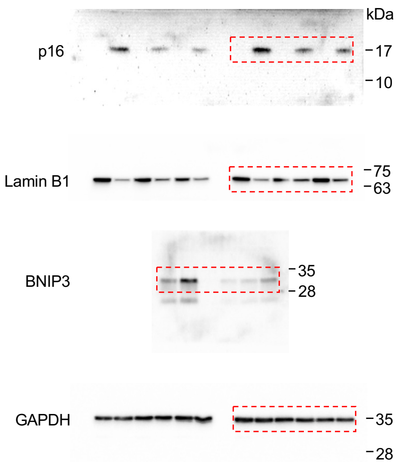

Fig. S2D

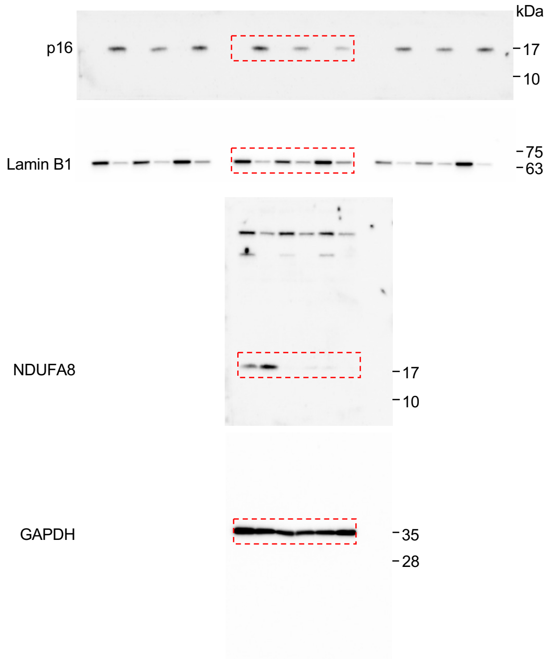

Fig. S3B

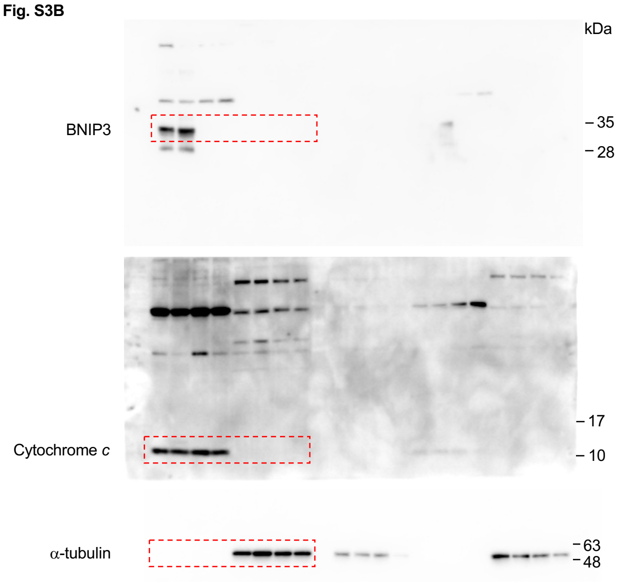

Fig. S4A

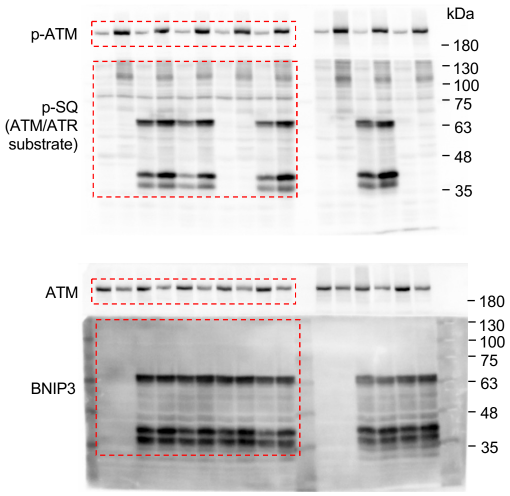

Fig. S4C

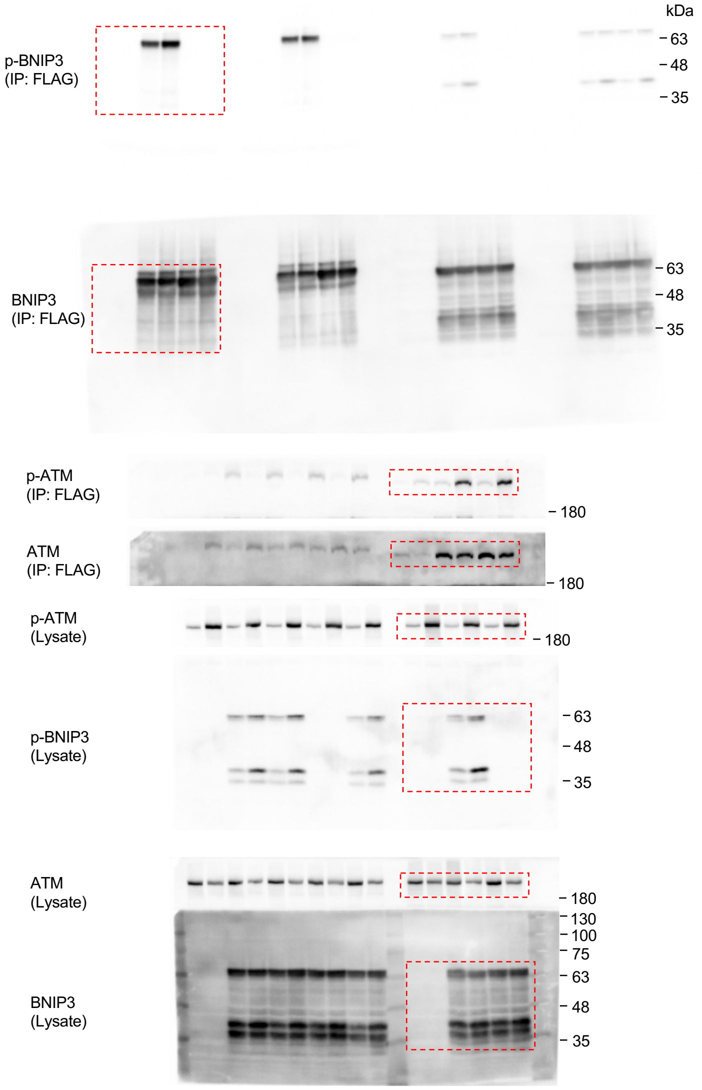

Fig. S4D

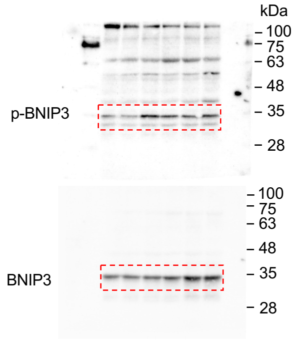

Fig. S4E

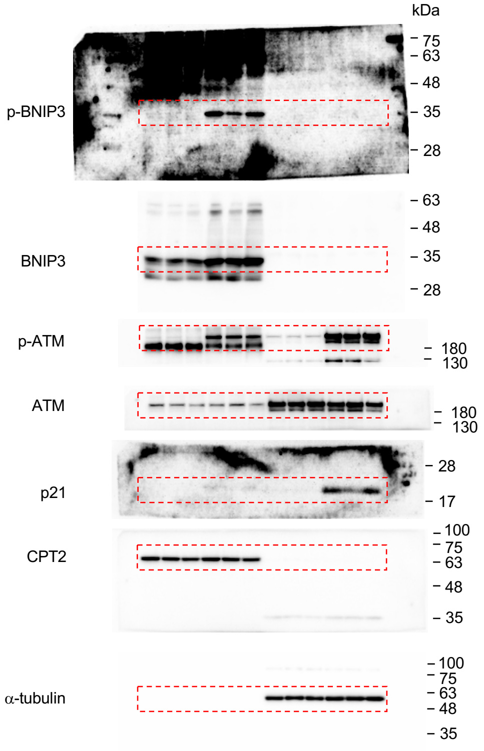

Fig. S4F

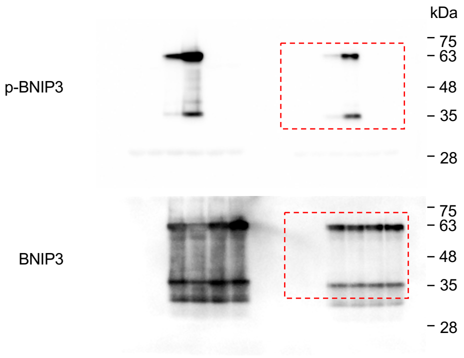

Fig. S5A

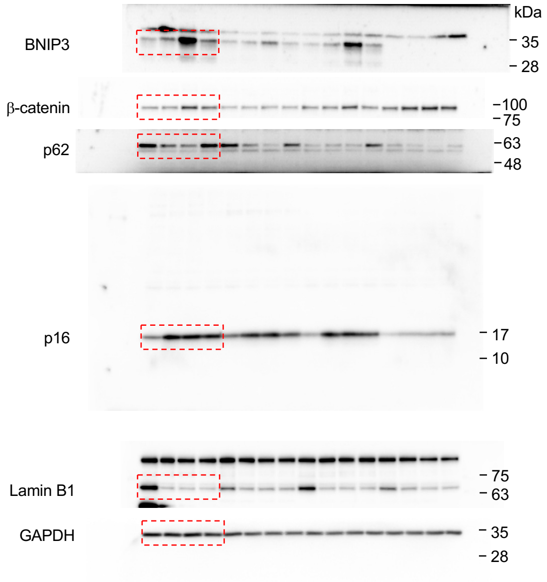

Fig. S5B

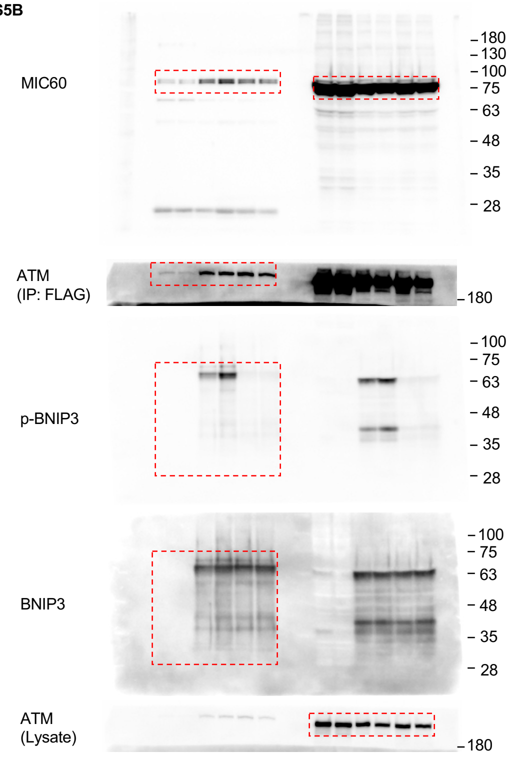

Fig. S5C

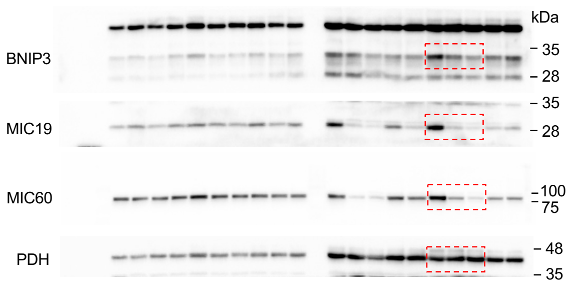

Fig. S5D

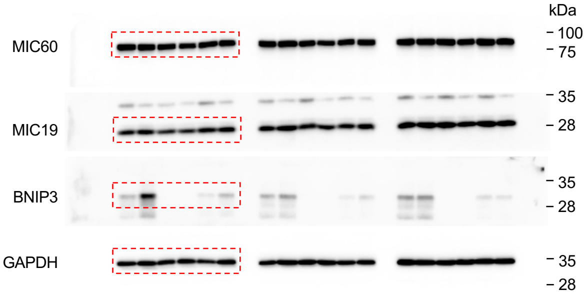

Fig. S9B

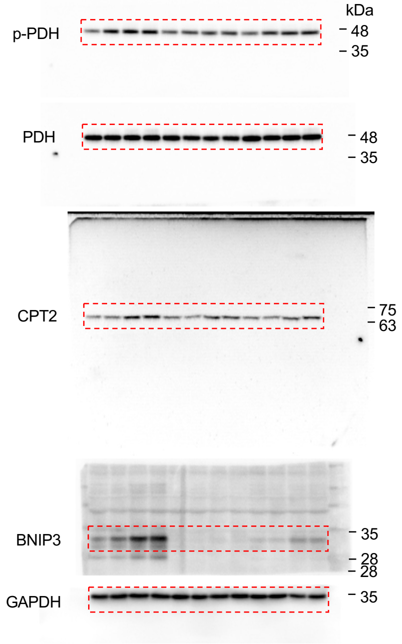

Fig. S9C

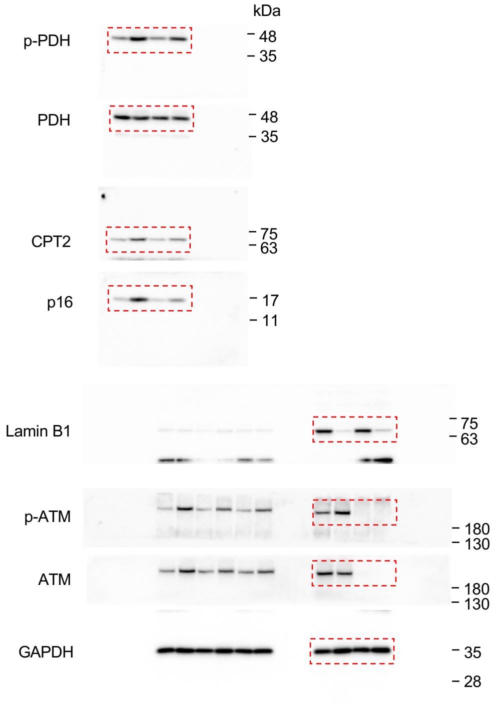

Fig. S9D

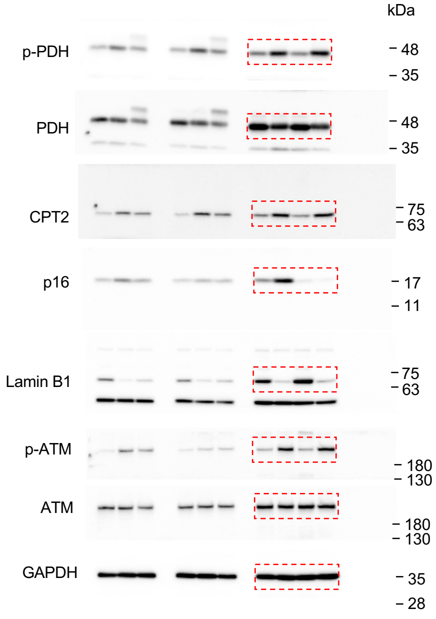

Fig. S11A

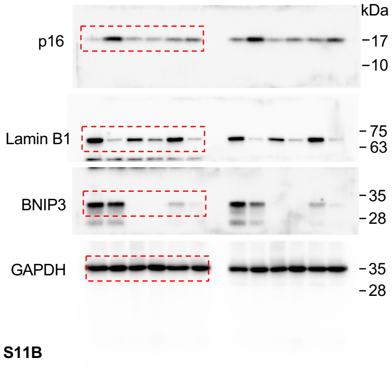

Fig. S11B

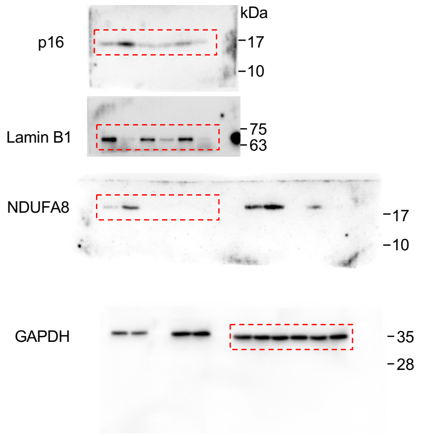

Fig. S11D

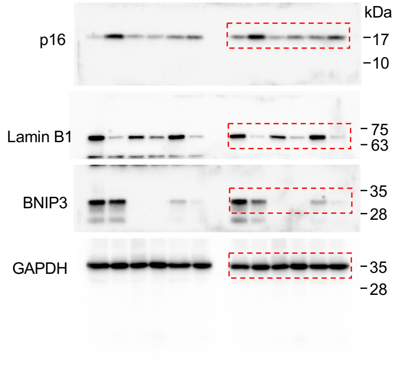

Fig. S11E

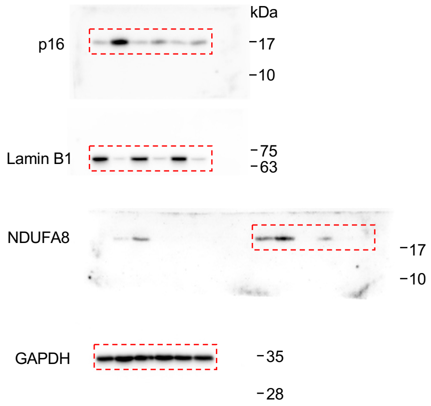

Fig. S12A

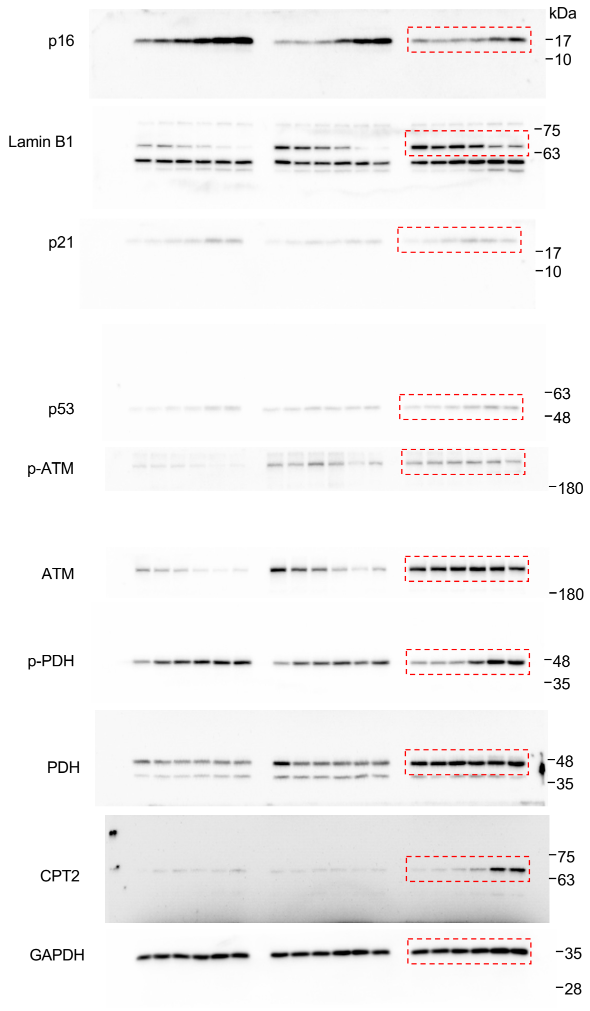

Fig. S12B

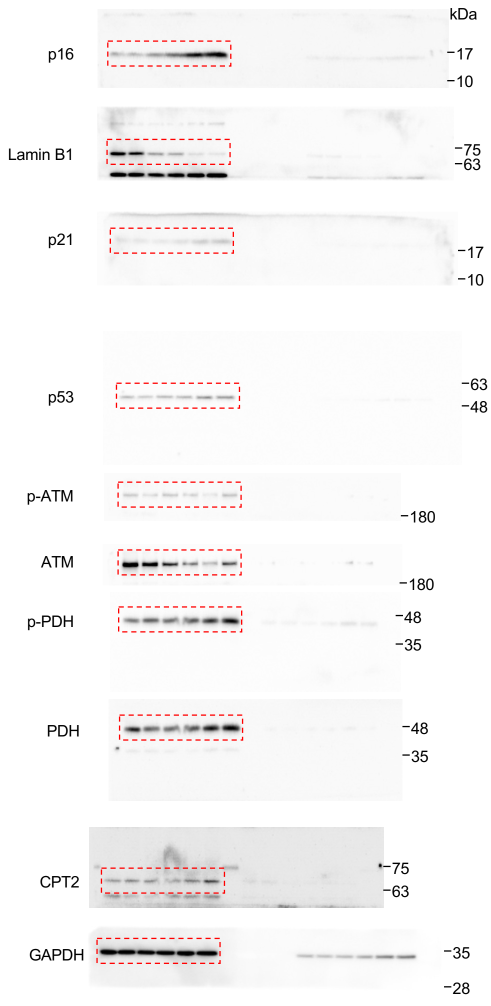

Fig. S12F

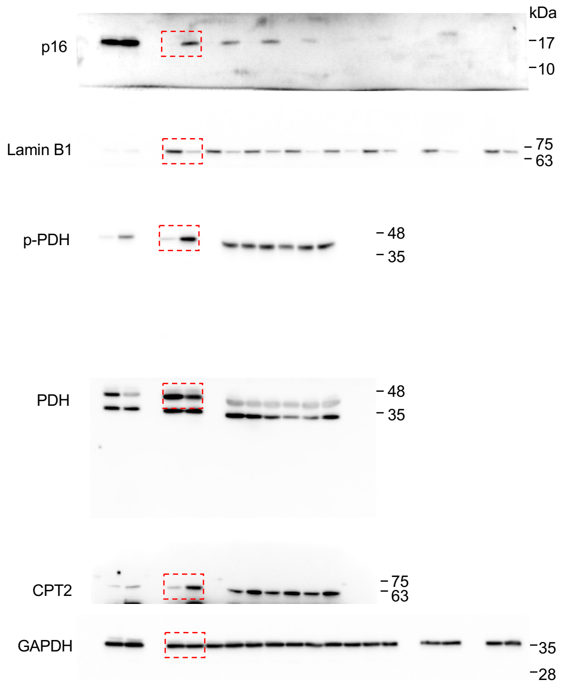

Fig. S12G

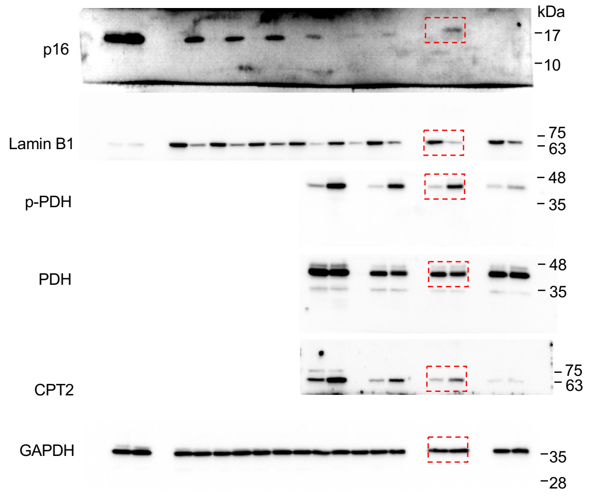

Supplement: Supplementary file 1 — Figs. S1 to S12 Uncropped gels Legends for tables S1 and S2 Legends for movies S1 to S4 [file sciadv.ado5887_sm.pdf]
